# The role of nanocerianite (CeO_2_) in the stability of Ce carbonates at low-hydrothermal conditions†

**DOI:** 10.1039/d3ra00519d

**Published:** 2023-02-28

**Authors:** Adrienn Maria Szucs, Melanie Maddin, Daniel Brien, Remi Rateau, Juan Diego Rodriguez-Blanco

**Affiliations:** a Department of Geology, School of Natural Sciences, Trinity College Dublin Ireland szcsa@tcd.ie; b iCRAG, Department of Geology, School of Natural Sciences, Trinity College Dublin Ireland

## Abstract

The formation of cerianite (CeO_2_) was investigated at low hydrothermal conditions (35–205 °C) *via* two experimental settings: (1) crystallisation from solution experiments, and (2) replacement of Ca–Mg carbonates (calcite, dolomite, aragonite) mediated by Ce-bearing aqueous solutions. The solid samples were studied with a combination of powder X-ray diffraction, scanning electron microscopy, and Fourier-transform infrared spectroscopy. The results revealed a multi-step crystallisation pathway: amorphous Ce carbonate → Ce-lanthanite [Ce_2_(CO_3_)_3_·8H_2_O] → Ce-kozoite [orthorhombic CeCO_3_(OH)] → Ce-hydroxylbastnasite [hexagonal CeCO_3_(OH)] → cerianite [CeO_2_]. We found that Ce carbonates can decarbonise in the final stage of the reaction, forming cerianite which significantly increases the porosity of the solids. The redox behaviour of Ce combined with the temperature, and the availability of CO_2_^3−^ govern this crystallisation sequence, the sizes, morphologies, and crystallisation mechanisms of the solid phases. Our results explain the occurrence and behaviour of cerianite in natural deposits. These findings also present a simple, environmental-friendly, and cost-efficient method for the synthesis of Ce carbonates and cerianite with tailored structures and chemistries.

## Abstract 2

Cerianite (CeO_2_) crystals obtained during synthesis in solution-based and solution-mediated replacement experiments.

## Graphical abstract

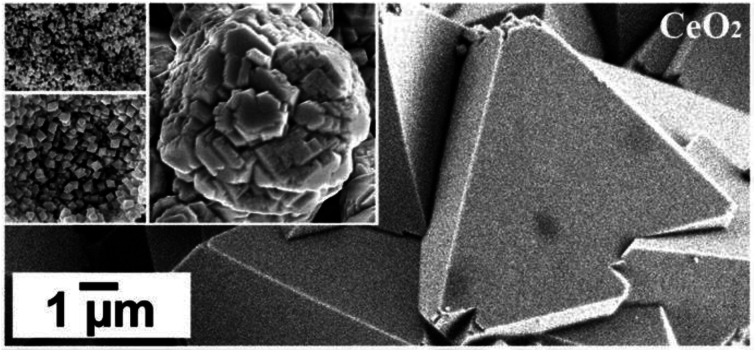

## 1. Introduction

Cerianite (CeO_2_) is a translucent, dark-green-amber-coloured mineral from the cubic crystal system with an octahedral morphology.^1^ It precipitates under hydrothermal oxidative conditions or forms as the oxidation product of Ce^3+^-bearing minerals^2^ and remains stable in neutral to alkaline pH conditions.^3^ Cerium can adjust its electron configuration best fitted to the environment; as an oxide, it can exist as Ce_2_O_3_ (with Ce^3+^) or CeO_2_ (with Ce^4+^).^4^ The result is the unique properties of Ce-oxide with a high oxygen storage capacity, optical properties, and malleable morphologies.^5–8^ Thus, Ce-oxide is used in various fields including catalysis,^5,9–11^ biological-,^12^ gas-sensing,^13^ solar cells,^14^ or glass polishing.^15^ It is also investigated in biomedical research due to its antioxidant properties;^11,16–20^ for example, cerium oxide nanoparticles are tested as therapeutic agents for the treatment of diseases associated with oxidative stress and inflammation, including cancer.^19,21,22^

Synthetic cerianite has been broadly studied with the goal of targeted material design for industrial purposes, thus, several cerianite synthesis methods have been developed; for example hydrothermal,^23,24^ solution combustion,^25^ reversed micelles,^26^ sol–gel,^27,28^ biosynthesis^19^ among other production methods. Cerianite can also be obtained from solution^25^ or from the interaction of carbonate minerals with Ce-bearing solutions.^29^ Synthesis conditions such as the pH, temperature, time, and method, govern the size, morphology, and surface properties of the final products which will define the behaviour and hence, the use and applications of the materials.^30^ Therefore, understanding the exact roles of the parameters involved in the synthesis is fundamental for designing the targeted materials. Although several settings and synthesis conditions are well-defined (Table ESI-1†), including the characteristics and usage of the final cerianite product, there is still a lack of understanding of the precise crystallisation mechanisms and pathways of cerianite.

Similarly, the same information gap remains about naturally occurring cerianite (Table ESI-1†). In nature, cerianite can be found in association with rare earth elements (REEs)-bearing carbonates (*e.g.*, bastnasite) and REE-phosphates (*e.g.*, monazite).^31–36^ It is identified as a late-stage secondary mineral.^2,31,37,38^ For example, A. Mahmoud and Williams-Jones^31^ suggested that cerianite is the product of the weathering of minerals containing light REEs such as monazite and bastnasite in their studied granitic batches from Egypt. Similarly, Zaitsev *et al.*^39^ found that cerianite formed during the alteration of apatite as a result of carbonate weathering precipitated from groundwater. Moore *et al.*^40^ reported cerianite in Bear Lodge carbonatite, Wyoming, USA, that partially replaced early-crystallised ancylite and fluorocarbonates; they also observed Ce-depleted monazite in association with cerianite at certain localities. Van Rythoven *et al.*,^38^ studied the same carbonatite and found that hydrothermally-altered carbonatite with unoxidized REEs occurs primarily as ancylite and parisite-synchysite, while monazite, bastnasite, and cerianite were labeled as subordinate minerals, broken down by supergene oxidation. This shows that although we have a certain understanding of cerianite formation in natural settings, the exact crystallisation mechanisms and influencing conditions are still not fully understood.

As cerianite has such a high industrial value, it is necessary to investigate its formation and the behaviour of Ce in natural/laboratorial systems. This knowledge can be used to shape and improve our current Ce-bearing material design techniques – both economically and environmentally – and thus, to further enchance the applications of this solid. Also, it is critical to understand the effect of geochemical conditions relevant to cerianite as currently, little is known of its environmental chemistry and thus, toxicity.^41^ Our experimental setting provides primary information on the synthesis of nano- and micro-sized Ce carbonate and oxide; these findings are particularly important as they advance the field of materials engineering and design, but also, aid the field of exploration, exploitation, and extraction of rare earths.

## 2. Methods

Two types of experimental methods were set up: (1) solution experiments and (2) replacement experiments. The combination of these two experimental settings and the studied temperature ranges allowed us to gain an in-depth picture on the kinetics and the governing factors during the crystallisation of Ce-bearing solids in the Ce–CO_3_–H_2_O system and provide detailed description for future material fabrication.

In the solution experiments, 10 ml of 50 mM Ce-bearing aqueous (Milli-Q) solutions (pH ≈ 5.1) were added to 10 ml of 50 mM Na_2_CO_3_ solutions in 20 ml Teflon-lined stainless-steel autoclaves at different temperatures (35, 50, 80 °C) and at saturated water vapor pressures (Table 1). To get more insight into the interaction of Ce and carbonate ions and the effects of temperature and concentration, additional 10 ml Ce-bearing aqueous (Milli-Q) solution (pH ≈ 5.1) mixed with 10 ml of Na_2_CO_3_ solution experiments were pre-heated and placed in hydrothermal reactors at 80 °C with different molar ratios of 1 : 1, 3 : 4 and 1 : 2 (Table 1). The solid samples were taken carefully at increasing time intervals. The reaction products were chilled to room temperature and filtered through 0.2 μm polycarbonate membranes by using a vacuum filtration unit. The solids were then placed into an oven at 50 °C for 30 min to remove any excess water.

**Table 1:** Experimental conditions, identities and morphologies of the solid Ce carbonate phases formed during the interaction of Na_2_CO_3_ solutions with Ce-bearing aqueous solutions

Experiment details	*T* (°C)	Time (days)	Phase formed	Morphology
**Solution experiments**				
10 ml, 50 mM REE + 10 ml 50 mM Na_2_CO_3_	35	1	58% Lan + 42% Koz	Lan: thin platy crystals
		2	<1% Lan + 98% Koz + <1% Cer	Koz: small prisms, elongated prisms
		8	92% Koz + 8% Cer	Cer: small octahedrons
		15	81% Koz + 19% Cer	
		21	70% Koz + 30% Cer	
		28	54% Koz + 46% Cer	
		36	100% Cer	
10 ml, 50 mM REE + 10 ml 50 mM Na_2_CO_3_	50	1	90% Koz + 10% Cer	Koz: small prisms, elongated prisms
		2	67% Koz + 33% Cer	Cer: small octahedrons
		5	10% Koz + 90% Cer	
		6	100% Cer	
10 ml, 50 mM REE + 10 ml 50 mM Na_2_CO_3_	80	1	100% Koz	Koz: small prisms, elongated prisms
		3	52% Koz + 39% Cer	Cer: small octahedrons
		6	6% Koz + 94% Cer	
		7	100% Cer	

**Pre-heated solution experiments**				
20 mM; 10 ml – pre-heated 80 °C Ce(NO_3_)_3_ + 20 mM; 10 ml Na_2_CO_3_ (1 : 1)	80	3	<1% Lan + 98% Koz + <1% Cer	Lan: thin platy crystals
				Koz: small prisms, elongated prisms
				Cer: small octahedrons
15 mM; 10 ml – pre-heated 80 °C Ce(NO_3_)_3_ + 20 mM; 10 ml Na_2_CO_3_ (3 : 4)		3	<1% Lan + 7% Koz + 92% Cer	Koz: small prisms, elongated prisms
				Cer: small octahedrons
10 mM; 10 ml – pre-heated 80 °C Ce(NO_3_)_3_ + 20 mM; 10 ml Na_2_CO_3_ (1 : 2)		3	41% Koz + 59% Cer	Koz: small prisms, elongated prisms
				Cer: small octahedrons

In the replacement setting, 0.1 g of calcite, dolomite, or aragonite with sizes of 0.5–1.0 mm were added to 50 ml of 50 mM Ce-bearing aqueous (Milli-Q) solutions (Ce(NO_3_)_3_·6H_2_O; pH ≈ 5.1). The solutions each were prepared using cerium(iii) nitrate hexahydrate (Ce(NO_3_)_3_·6H_2_O) reagents (Sigma-Aldrich; 99.99% trace metals basis). The solutions and solids were placed in 50 ml Teflon-lined stainless-steel autoclaves at different temperatures (50, 90, 165, and 205 °C) and saturated water vapor pressures (Tables 2 and 3). Solid samples were taken carefully at increasing time intervals which were then placed into an oven at 50 °C for 30 min to remove any excess water. Also, control experiments consisting only of Ce-bearing aqueous solutions in closed reactors were carried out at the same temperatures of the solution and replacement experiments.

**Table 2:** Experimental conditions, identities and morphologies of the solid Ce carbonate phases formed during the interaction of calcite grains with Ce-bearing aqueous solutions

Calcite				
*T* (°C)	Time (days)	% phase consumed	Phase formed	Morphology
50	1	<1	Koz	Koz: small prisms, elongated prisms
	2	8		Cer: small octahedrons
	3	17		
	10	67		
	14	75		
	22	93	92% Koz + <1% Cer	
	29	100	99% Koz + <1% Cer	
	44	100	99% Koz + <1% Cer	
90	1	10	10% Koz	Koz: small prisms, elongated prisms
	2	21	21% Koz	Cer: small octahedrons
	5	31	31% Koz	
	11	94	94% Koz	
	14	97	96% Koz + <1% Cer	
	18	100	99% Koz + 1% Cer	
	25	100	98% Koz + 2% Cer	
	33	100	97% Koz + 3% Cer	
	38	100	96% Koz + 4% Cer	
165	1	21	18% Koz + 2% HB + <1% Cer	Koz: small prisms, elongated prisms
	2	64	6% Koz + 3% HB + 55% Cer	HB: triangular prisms
	7	100	Cer	Cer: small octahedrons
205	1	100	Cer	Cer: small octahedrons

**Table 3:** Experimental conditions, identities and morphologies of the solid Ce carbonate phases formed during the interaction of dolomite and aragonite grains with Ce-bearing aqueous solutions

*T* (°C)	Time (days)	Dolomite			Aragonite		
		% phase consumed	Phase formed	Morphology	% phase consumed	Phase formed	Morphology
50	1	<1	Koz	Koz: small prisms, elongated prisms	4	3% Lan + 1% Koz	Lan: thin platy crystals, Koz: small prisms, elongated prisms
	3	18			6	2% Lan + 4% Koz	
	7	27			23	2% Lan + 21% Koz	
	14	35			46	5% Lan + 41% Koz	
	21	45			56	3% Lan + 53% Koz	
	28	57			70	2% Lan + 68% Koz	
	42	78			100	Koz	
	56	100					
80	1	35	Koz	Koz: small prisms, elongated prisms, Cer: small octahedrons	34	Koz	Koz: small prisms, elongated prisms, Cer: small octahedrons
	3	47			41		
	7	53			62		
	14	71	70% Koz + <1% Cer		69		
	21	82	81% Koz + <1% Cer		89	89% Koz	
	28	93	92% Koz + <1% Cer		95	94% Koz + <1% Cer	
	42	100	98% Koz + 2% Cer		100	99% Koz + <1% Cer	
	56	100	97% Koz + 3% Cer		100	99% Koz + <1% Cer	
165	3	39	<1% Koz + 4% HB + 34% Cer	Koz: small prisms, elongated prisms	58	29% Koz + 25% Cer + 4% HB	Koz: small prisms, elongated prisms
	7	99	<1% Koz + 11% HB + 88% Cer	HB: triangular prisms	87	7% HB + 80% Cer	HB: triangular prisms
	14	100	Cer	Cer: small octahedrons	100	Cer	Cer: small octahedrons
205	1	100	Cer	Cer: small octahedrons	100	Cer	Cer: small octahedrons

### 2.1. Fourier-transform infrared spectroscopy

A Nicolet Summit FTIR Spectrometer with an Everest Diamond ATR Accessory was used for obtaining a FTIR pattern of the sample, using a wavelength range of 4000–650 cm^−1^, and the resolution is 0.6 cm^−1^. OMNIC Paradigm Desktop Software was used for material characterisation, identification, and verification.

### 2.2. Powder X-ray diffraction

Powder X-ray diffraction (XRD) was used to identify and quantify any newly formed crystalline compounds. The solids obtained in the solution experiments did not require additional grounding, but the replacement reaction experiments were first grounded and then analysed by XRD; using a Bruker D5000 X-ray diffractometer (Cu Kα radiation, 0.01° per step from 5 to 70° 2*θ* at 1° min^−1^). The XRD patterns were identified with Diffract Suite EVA software from Bruker in combination with the Powder Data File (PDF-4, The International Centre for Diffraction Data). Rietveld refinement software (TOPAS)^42^ was used to conduct pattern-matching refinement and quantification of the crystalline phases; no further pre-processing of data (normalisation, *etc.*) was needed. Crystallite sizes were calculated from the Bragg peak full-width half-maximum (FWHM) using the Scherrer equation^43^ and the unit cell parameters were determined with TOPAS.^42^

### 2.3. SEM

Scanning electron microscopy (SEM) was used to characterise the precipitates from the solution experiments, the changes in the morphology of the host minerals (calcite, dolomite, or aragonite), and the newly formed crystalline phases. Samples from the replacement experiments were carbon-coated and placed into a Tescan MIRA4 S8000 FEG-SEM operating under high-vacuum conditions and equipped with four Oxford Instruments NanoAnalysis X-Max 170 mm^2^ EDS detector running Oxford Instruments NanoAnalysis AZtecTimed analysis software. Powders from solution experiments were Au–Pd coated and imaged with Tescan TIGER MIRA3 FEG-SEM equipped with two Oxford Instruments X-Max 150 mm^2^ EDS detectors running Oxford Instruments AZtec software. All analyses were performed using an accelerating voltage of either 5 or 10 kV for detailed imaging.

### 2.4. PHREEQC

The saturation indices (SI) of Ce-bearing carbonates during the equilibration of the Ce-bearing aqueous solutions with respect to calcite, dolomite, or aragonite were calculated with the hydrogeochemical code PHREEQC^44^ using the LLNL database^45^ The saturation index is defined as:
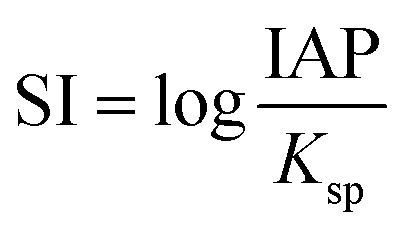
where IAP corresponds to the ion activity product in solution and *K*_sp_ is the solubility product of the solid phase. The solubility products of Ce-bearing carbonates were determined by Essington and Mattigod.^46^

## 3. Results

### 3.1. The solution experiments

**Fig. 1:**
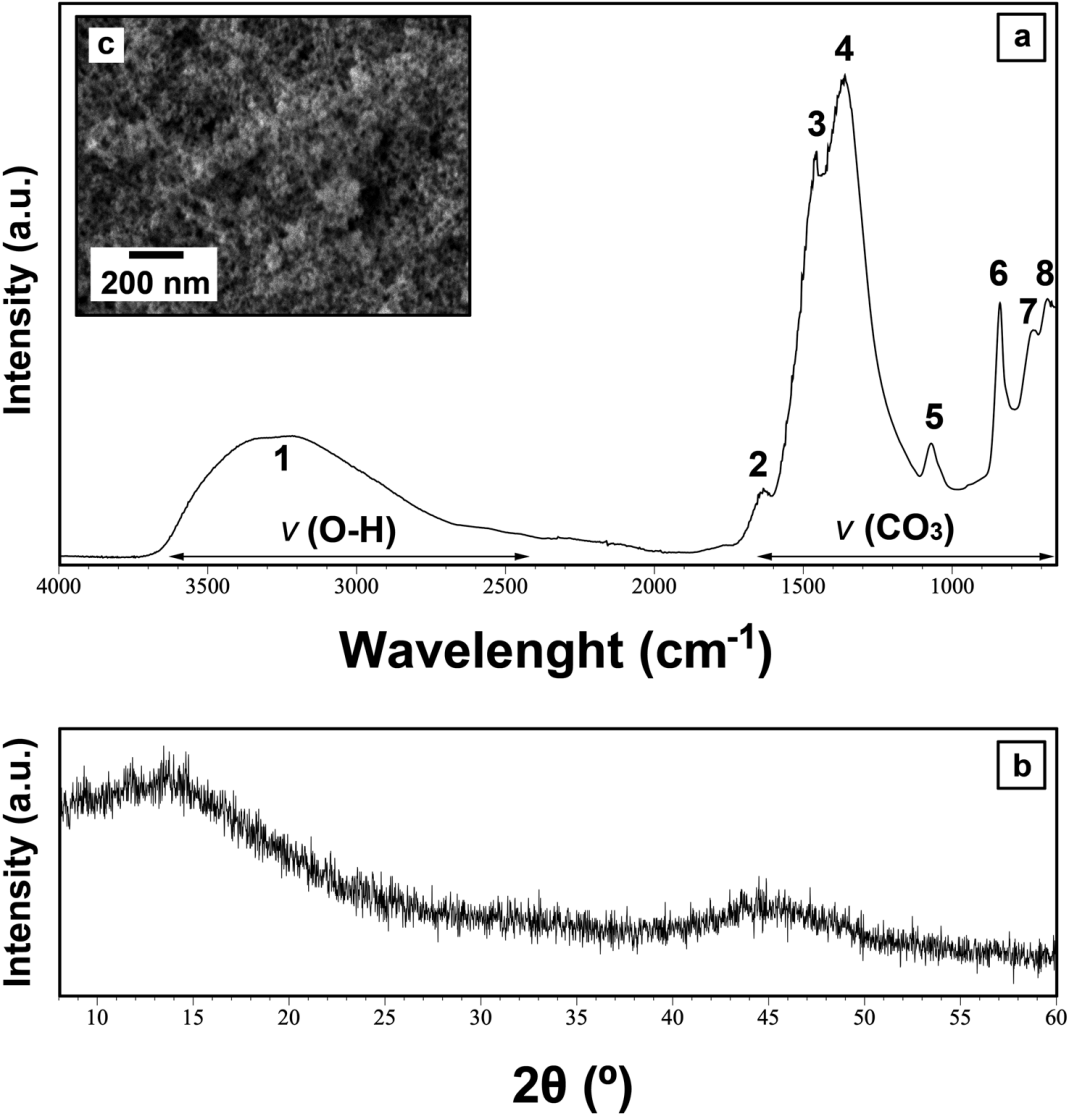
Characterization of the primary Ce carbonate formed during the crystallization from solution experiments at ambient temperature. (a) Representative XRD pattern of the initial Ce-precipitate; (b) FTIR spectra of the initial Ce-precipitate showing the large OH band (1) and the main carbonate bands (2–6). Details of the band assignments are presented in Table ESI-2† and discussed; (c) secondary electron SEM image of the initial Ce-precipitate.

Upon mixing of the Ce(NO_3_)_3_·6H_2_O and the Na_2_CO_3_ starting solutions at all applied temperatures within the first hour, a white precipitate formed, respectively. The XRD patterns of these materials showed only two humps centred at ∼10–15 and 45° 2*θ* (Fig. 1a), indicating the presence of poorly-ordered materials. FTIR analysis of this material revealed the typical carbonate and O–H vibrations (Fig. 1b). The broad band between ∼2500 and 3700 cm^−1^ (marked as band 1; detailed assignments, see Table ESI-2†) represents O–H stretching vibrations from structural water. The most intense vibrations were located between ∼1455 and 679 cm^−1^ (bands 2–8; detailed assignments, see Table ESI-2†) and are characteristic of the main stretching vibrations of the carbonate ions. The pattern is similar to the La–Nd-amorphous precursors described by Vallina *et al.*^47^ This confirms the presence of a strongly hydrated and amorphous Ce carbonate, similar to the well-known hydrated amorphous carbonates produced in the laboratory or found in nature (*e.g.*, amorphous Ca–Mg carbonates).^48–54^Fig. 1c shows the SEM image of this material, revealing the usual nano-spherular morphology of amorphous carbonate precursors.

The pre-heated mixture of Ce(NO_3_)_3_·6H_2_O and Na_2_CO_3_ solutions to 80 °C with three different concentration ratios, sampled after 3 hours (Table 1), resulted in the formation Ce-lanthanite [Ce_2_(CO_3_)_3_·8H_2_O] (PDF 00-038-0377), Ce-kozoite [Ce(CO_3_)OH] (isostructural with La-kozoite; PDF 00-049-0981) and nano-crystalline cerianite [CeO_2_] (PDF 00-043-1002), the latter seen as broad peaks in the XRD patterns shows (Fig. ESI-1†). Unit cell parameters of these crystalline phases are reported in Table ESI-3.† Phase quantification using Rietveld refinement revealed that the experiments with the molar ratio of 1 : 1 resulted in the formation of Ce-kozoite, <1% Ce-lanthanite and <1% cerianite. However, decreasing the concentration of Ce(NO_3_)_3_·6H_2_O in the solution, increased the production of cerianite (Table 1). These results indicate that the reaction pathway is strongly dependent on the Ce/CO_3_ ratio in the solution.

**Fig. 2:**
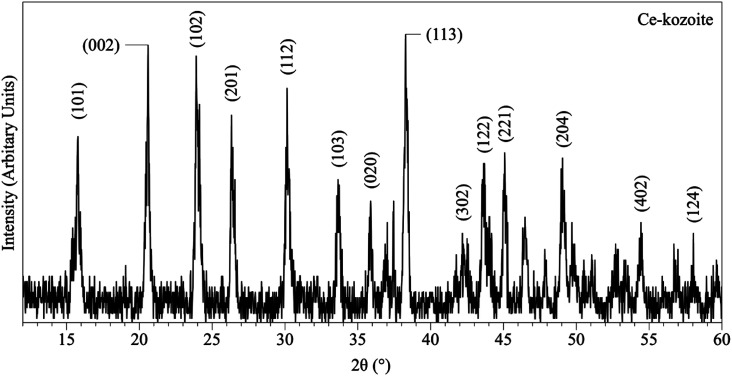
Representative XRD pattern of Ce-kozoite, produced at 80 °C after 1 day in the solution experiments.

**Fig. 3:**
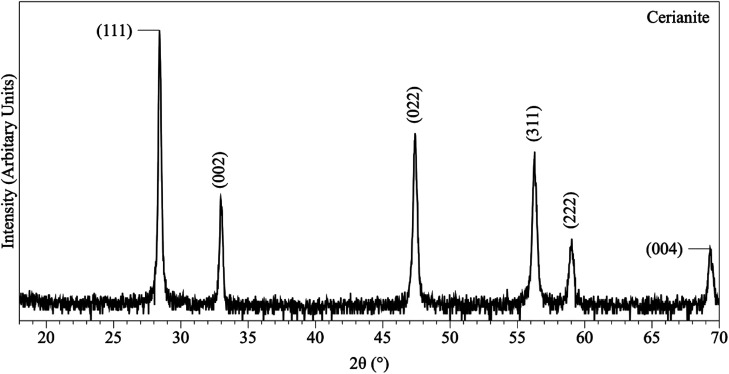
Representative XRD pattern of cerianite, produced at 80 °C after 7 days in the solution experiments.

**Fig. 4:**
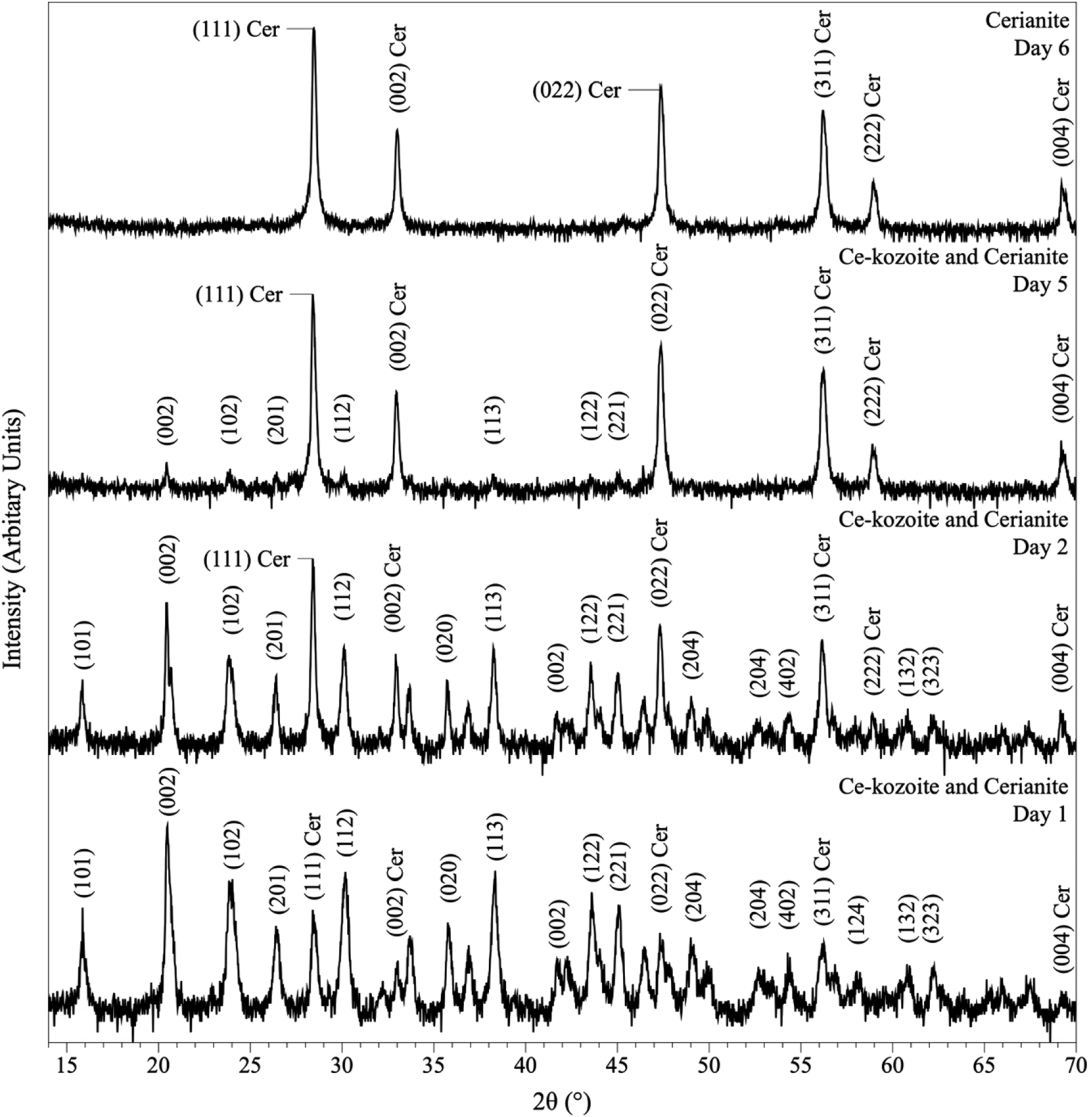
Powder XRD patterns showing the solid phases obtained in the solution-based experiments at 50 °C after 1, 2, 5 and 6 days of reaction, showing the transformation of Ce-kozoite into cerianite.

In the experiments placed at 35, 50, and 80 °C and sampled at increased intervals (Table 1) three crystalline materials were detected: Ce-lanthanite, Ce-kozoite and cerianite (Table 1). Control experiments at these temperatures showed no sign of solid precipitation. The XRD pattern of Ce-kozoite and cerianite are presented in Fig. 2 and 3, and an SEM image showing Ce-lanthanite is presented in Fig. ESI-2.† Ce-lanthanite occurred with notable quantity in the experiment at 35 °C in <48 hours of reaction. Ce-kozoite formed on each examined temperature at the earlier stages of the reactions. Similarly, cerianite was found at all temperatures; however, its weight% increased at the later stages of the experiments and with temperature. The reaction pathway was time-dependent: Ce-lanthanite formed in the first 24 hours of the 35 °C experiments, alongside Ce-kozoite. Within 48 hours, Ce-kozoite dominated the sample with 98 weight% in the presence of <1 weight% Ce-lanthanite and <1 weight% cerianite. With time, the presence of Ce-kozoite decreased while the quantity of cerianite increased. Cerianite remained stable after 36 days onwards. At 50 and 80 °C, the reaction proceeded similarly but faster; Ce-kozoite fully transformed into cerianite within 6 days and remained stable onwards. The evaluation of the XRD patterns of cerianite also revealed an increase of crystallite size with time from 13 to 410 nm. Fig. 4 shows the transformation of Ce-kozoite into cerianite in 6 days at 50 °C.

**Fig. 5:**
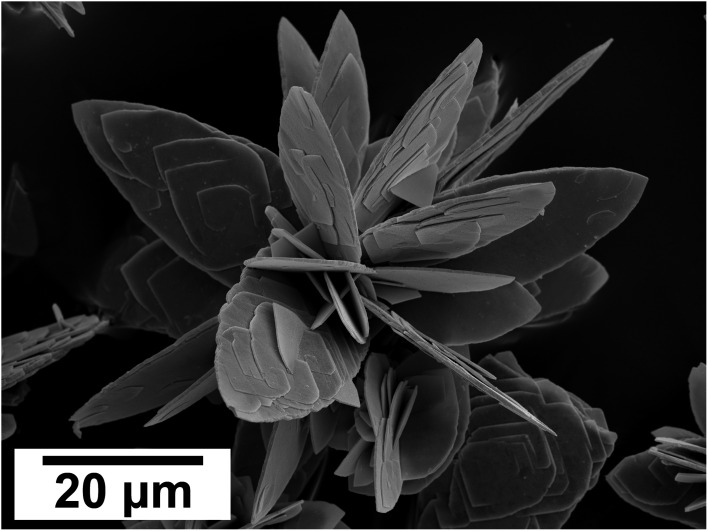
Secondary electron SEM image of Ce-lanthanite obtained in the solution-based experiments at 35 °C, after 1 day of reaction.

**Fig. 6:**
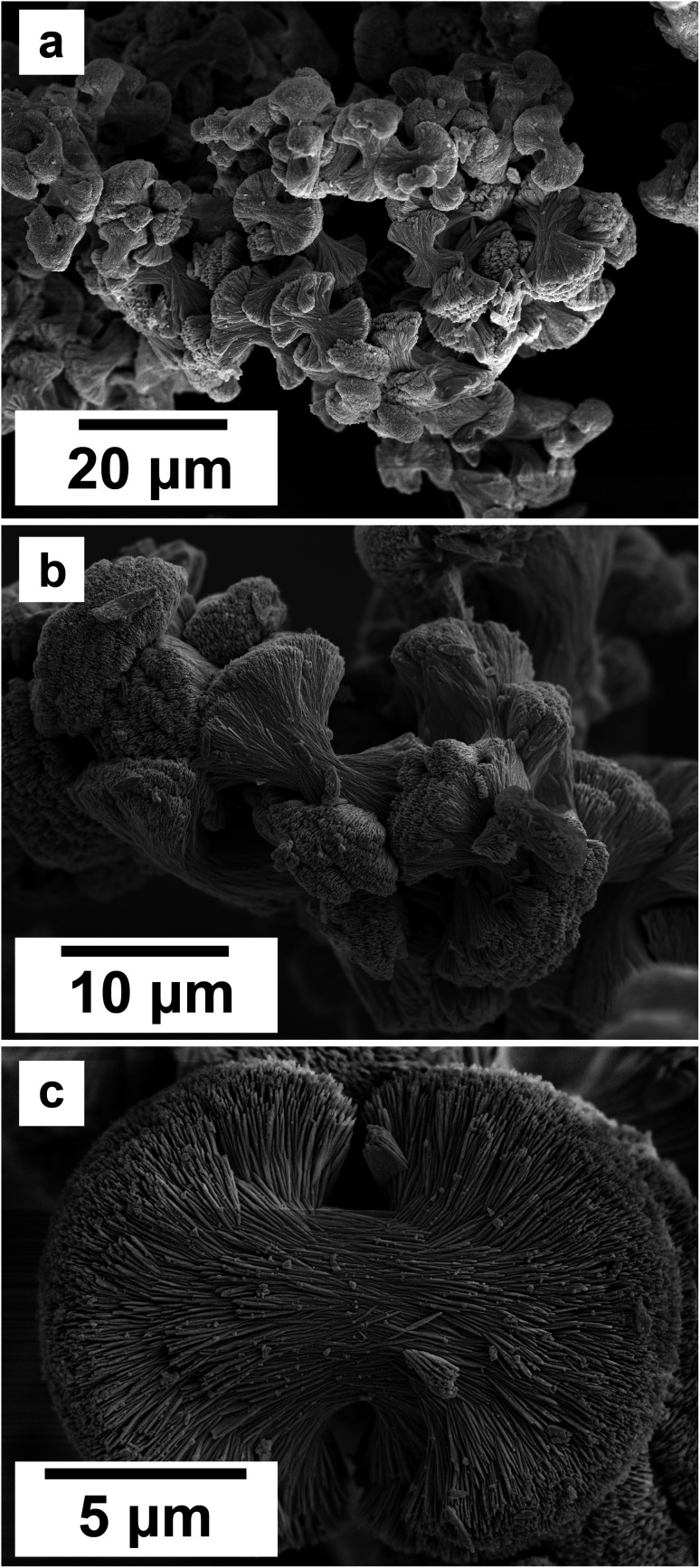
(a–c) Secondary electron SEM images of Ce-kozoite crystallised in the solution experiments preheated at 80 °C.

**Fig. 7:**
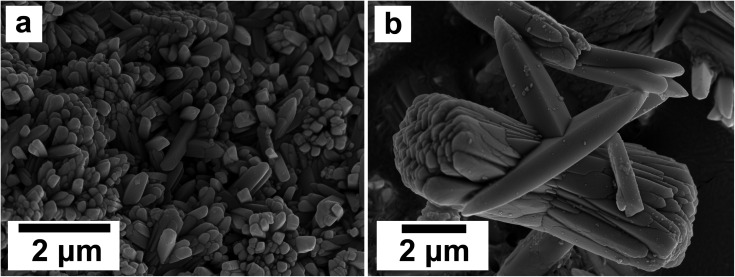
Secondary electron SEM images of bladed or spindle-shaped Ce-kozoite crystals (a and b) formed from solution at 35 °C.

**Fig. 8:**
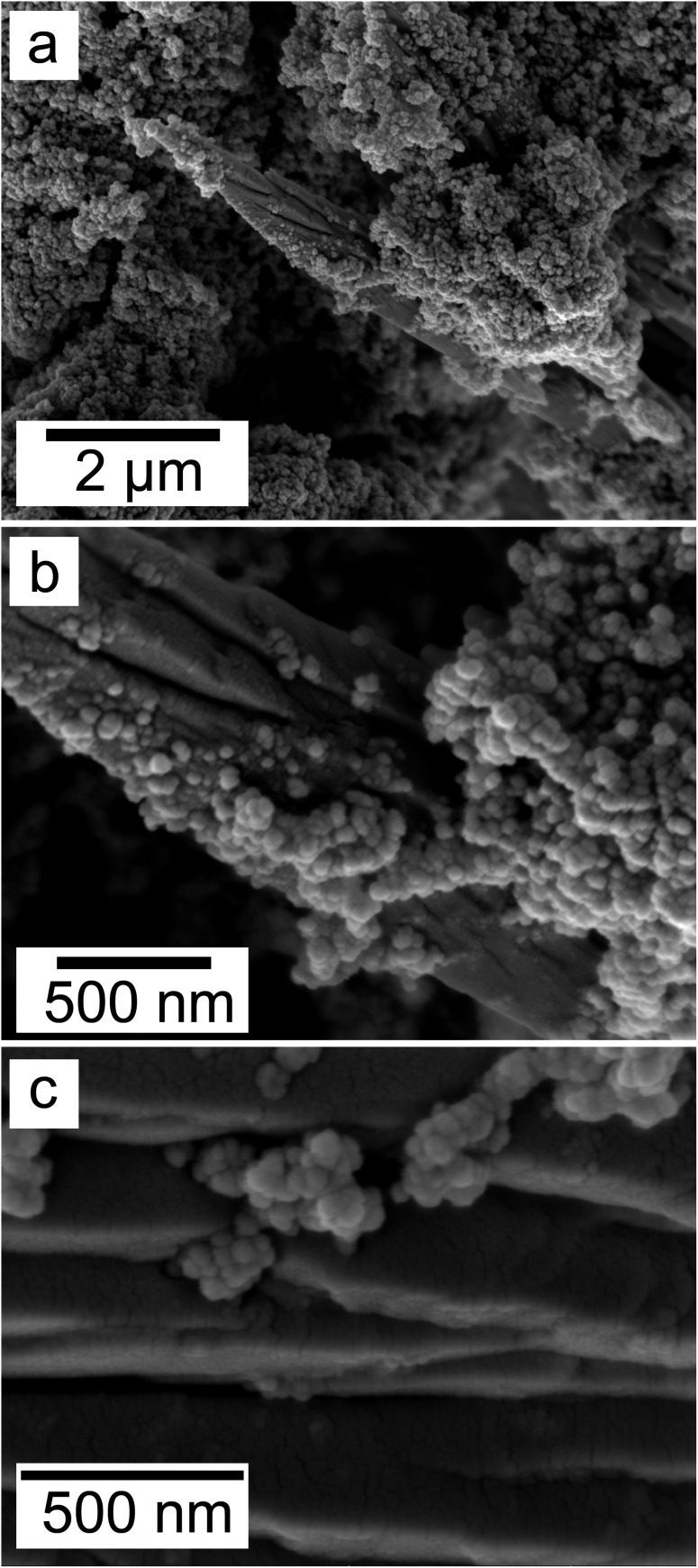
Secondary electron SEM images (a–c) of nanosized cerianite nanocrystals formed on the surface of Ce-kozoite at 35 °C, in the solution experiments.

**Fig. 9:**
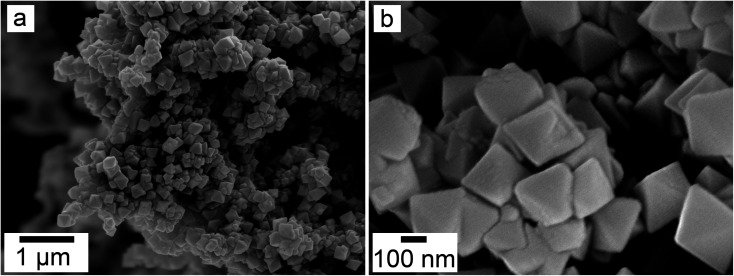
Secondary electron SEM images (a and b) of cerianite octahedra crystallised at 35 °C from solution.

SEM revealed the morphologies of these crystalline materials: Ce-lanthanite formed euhedral shapes of ∼50–100 μm with crystal imperfections and twinning (Fig. 5). Ce-kozoite from solutions resulted in two different morphologies: dumbbell-shaped aggregates (∼10 μm) built by sheets (1 μm length, 50–100 nm thickness) formed in the preheated 1 : 1 experiment at 80 °C (Fig. 6), while the rest of the solution experiments resulted in more bladed or spindle-shaped Ce-kozoite crystals (5 μm length, 500 nm thickness), sometimes forming aggregates (Fig. 7). Cerianite occurred as ∼50–100 nm nanocrystals (Fig. 8 and ESI-3†), which formed directly from the solution or on the surface of Ce-kozoite. Later on, these nanocerianite crystals grew into more defined triangular pyramids and octahedrons with a size range between 100–200 nm (Fig. 9).

### 3.2. Interaction of Ca–Mg carbonate seeds with Ce-bearing solutions

The interaction of carbonate seeds (calcite, aragonite, dolomite) with Ce-bearing solutions resulted in the formation of a series of surface precipitates. Upon opening the reactors, the carbonate seeds were covered by a reddish surface precipitate. It is worth noting here that in the experiments placed above 165 °C some gas bubbles were observed on the walls of the reactors (Fig. ESI-4†). XRD analysis of the solids taken at different time intervals revealed a progressive replacement of the host grains by Ce-bearing solids with time (Tables 2 and 3). The combination of powder XRD and SEM techniques allowed the identification and quantification of the replacing materials, as well as the interpretation of the mechanisms of surface precipitation.

**Fig. 10:**
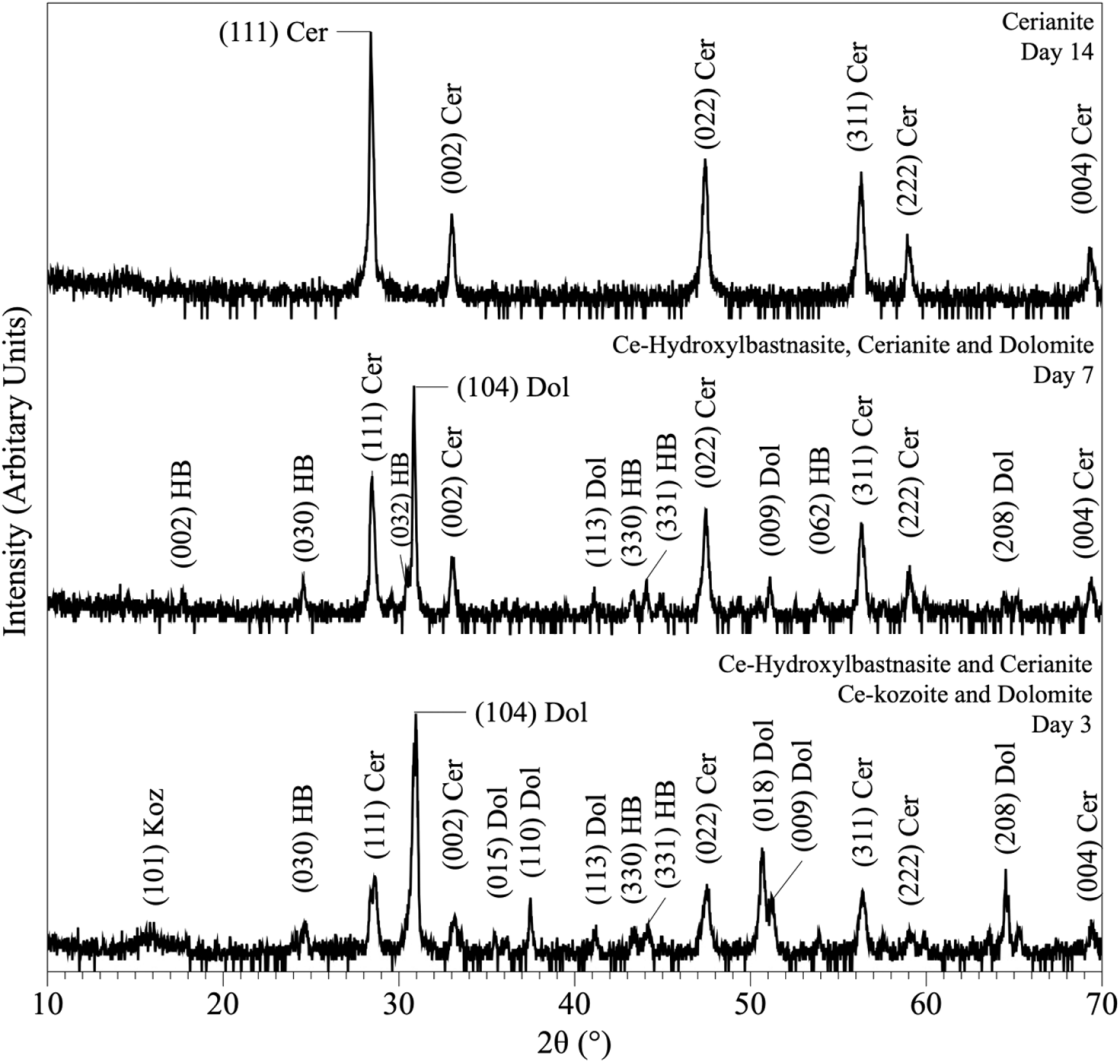
Powder XRD patterns showing the results of dolomite replacement experiments at 165 °C after 3, 7 and 14 days.

XRD revealed the formation of Ce-lanthanite, Ce-kozoite, Ce-hydroxylbastnasite [CeCO_3_(OH)] (PDF 00-017-0503), and cerianite (Fig. 2, 3, 4, and 10; ESI 3 and 4†). All phase quantification using Rietveld refinement are summarized in Tables 2 and 3. The control experiments placed at ≥165 °C resulted in the formation of a precipitate identified by XRD as cerianite. These revealed that the kinetics of crystallisation of the secondary REE-bearing minerals and the polymorph selection were controlled by temperature and the structure and chemistry of the host carbonate crystal. Regardless of the composition of the newly formed crystals, all host grains were fully replaced by crystalline Ce-bearing minerals at temperatures >50 °C. The kinetics of these replacement reactions were temperature-dependent and was found to be affected by the structure and chemistry of the host seeds. The experiments with calcite reached full-replacement by Ce-carbonates the fastest, while dolomite-hosted experiments required the longest time: for example, at 50 °C, calcite seeds were fully replaced within 29 days, while aragonite needed 42 days, and dolomite the longest 56 days, respectively.

At 50 °C, all experiments were translated into the replacement of the host crystal by Ce-kozoite, although in the experiments with aragonite a small amount Ce-lanthanite was also identified through the first 28 days of the reaction (Fig. ESI-5†), which subsequently transformed into Ce-kozoite, remaining stable afterwards. Calcite-hosted experiments resulted in the crystallisation of Ce-kozoite and <1% of cerianite by the end of the reaction. The reactions at 90 °C proceeded similarly but faster and the amount of cerianite was slightly more (<4% of cerianite) compared to the 50 °C experiments. At 165 °C, Ce-kozoite and Ce-hydroxylbastnasite had formed in < 1 day with calcite and in < 7 days with dolomite and aragonite (Fig. 10). The calcite grains were completely replaced by cerianite after 7 days while the dolomite and aragonite grains needed 14 days to be fully replaced. Above 205 °C, all experiments were completed and fully replaced by cerianite in <1 day.

**Fig. 11:**
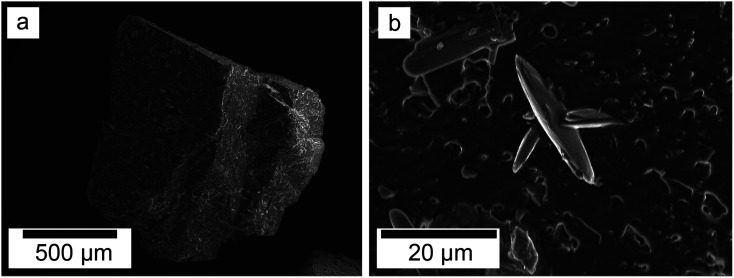
Secondary electron SEM images of (a) a dolomite seed (b) surface after 1 day at 50 °C showing dissolution marks and newly formed Ce-kozoite crystals.

**Fig. 12:**
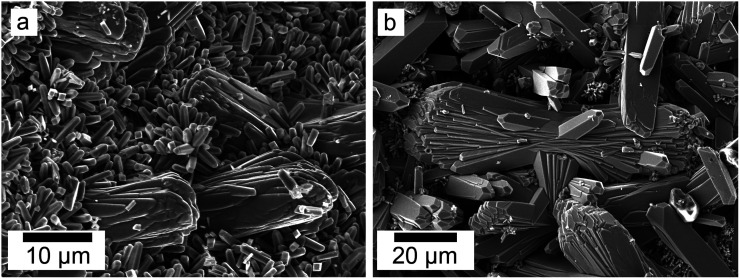
Secondary electron SEM images (a and b) of Ce-kozoite forming on the surface of dolomite grains at 50 °C.

**Fig. 13:**
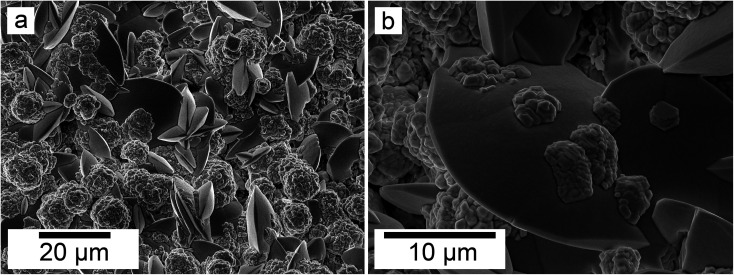
Secondary electron SEM images (a and b) of spindle-shaped Ce-hydroxylbastnasite and aggregates of nanocerianite crystals (indicated with the arrow) that formed on the surface of aragonite at 165 °C.

**Fig. 14:**
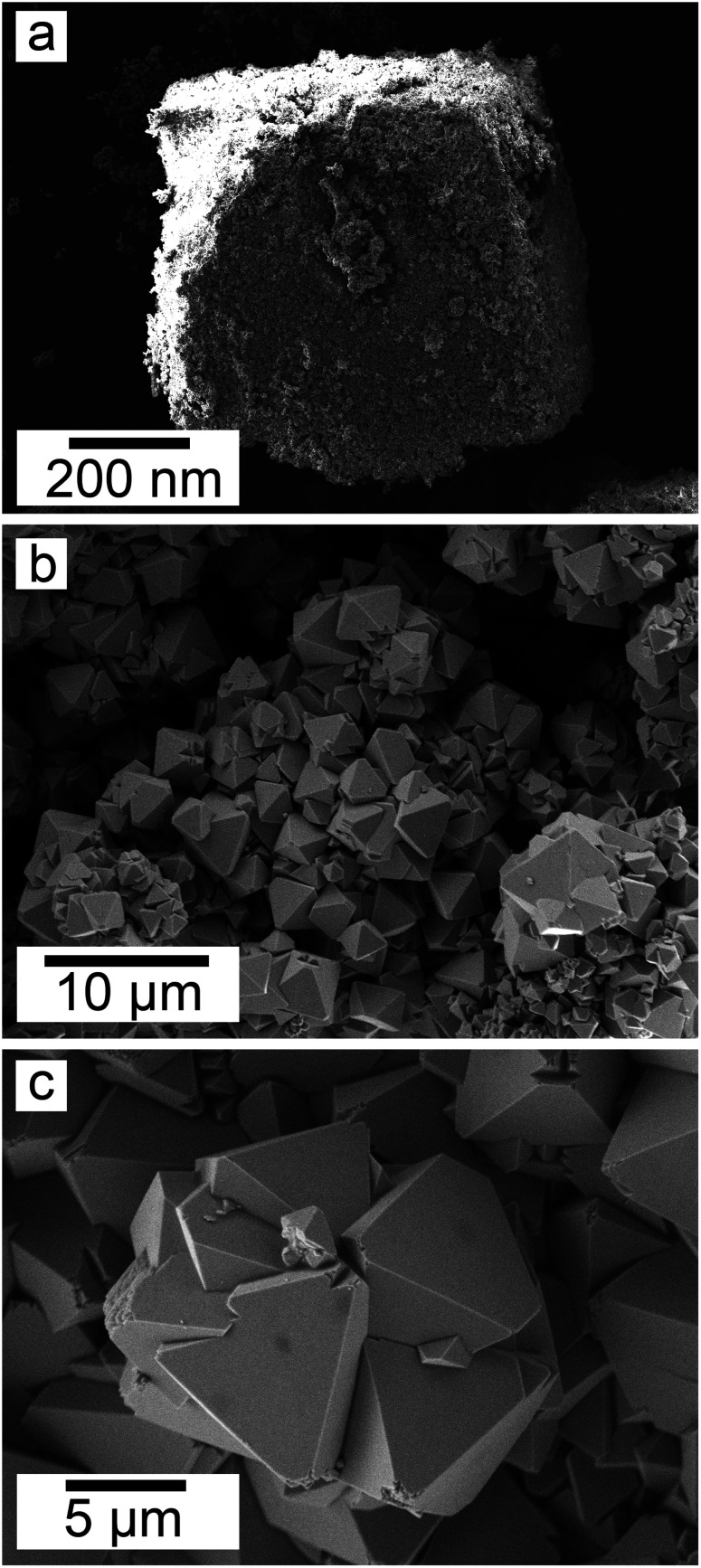
Secondary electron SEM images of (a) a dolomite pseudomorph fully replaced by cerianite at 165 °C, (b) cerianite up-close formed on the surface of a dolomite grain at 165 °C and (c) cerianite formed on an aragonite grain at 165 °C.

SEM revealed the formation of surface precipitates on calcite, dolomite, and aragonite grains in <1 day. A representative example is shown in Fig. 11, presenting a typical dolomite grain surface with precipitate after 1 day of reaction at 50 °C. Fig. 11b show signs of dissolution and small (∼20 μm) individual slightly elongated crystals started to cover the host grain. Afterwards, the crystals became more defined, covering the full surface of the host seed and hiding the dissolution marks of the host mineral. The final result was a pseudomorphic replacement in which the original morphology and dimensions of the host grains remained constant, but the host mineral was fully replaced by the newly formed Ce-bearing phases, supported by the results of XRD analysis. These newly formed phases had distinguishable morphologies: Ce-kozoite formed bladed and spindle-shaped morphologies with sizes ranging between 10 and 100 μm (Fig. 12), similar to solution experiments, but with larger crystals. Ce-hydroxylbastnasite crystallised as triangular prisms with sizes usually smaller than 15 μm (Fig. 13). Cerianite grew in the shape of octahedra with ∼2–5 μm size (Fig. 14), sometimes consisting of spheroidal aggregates of ∼10 μm size (Fig. 13 and 14). These cerianite end products had crystallite sizes ranging from 55 nm (spheroidal aggregates) and 300–450 nm (octahedra). The replacement of the host mineral by cerianite was translated into the development of very porous crystal pseudomorphs (*e.g.*, Fig. 14a). EDS analyses did not reveal any impurities in any of the newly formed solids.

## 4. Discussion

The combination of crystallisation from solution and replacement experiments revealed the behaviour of Ce in the presence of carbonate ions under low-hydrothermal conditions. In the solution experiments, the homogenous nucleation was a result of the interaction of Ce^3+^ and CO_3_^2−^ ions in the solution, forming different Ce-bearing precipitates at high supersaturation conditions. In the replacement experiments, the interaction of the carbonate host grain and the Ce-bearing solution resulted in the initial dissolution of the host and the release of Ca^2+^ and CO_3_^2−^ (and Mg^2+^ in the case of dolomite grains) into the solutions. This increased the supersaturation levels especially closer to the surface of the carbonate seeds, allowing the CO_3_^2−^ ions to interact with Ce^3+^ ions which led to the formation of surface precipitate of different Ce-bearing precipitates on the surface of the host grain.^55,56^ In all cases, the host carbonate grains were gradually covered by Ce-bearing surface precipitates and with time they were fully replaced periphery inwards. In both types of experiments, the reaction resulted in the formation of crystalline Ce-lanthanite, Ce-kozoite, Ce-hydroxylbasnasite and cerianite.

Taking all the replacement and solution experiments as a whole (Tables 1–3) we suggest the following multi-step crystallisation sequence: amorphous Ce carbonate → Ce-lanthanite → Ce-kozoite → Ce-hydroxylbastnasite → cerianite. However, the reaction pathway is strongly temperature dependent and does not proceed through some of the intermediate (metastable) steps, especially at high (165–205 °C) temperatures.

### 4.1. The precursor phase: amorphous Ce-carbonate

Amorphous Ce-carbonate was only identified in the solution experiments (Fig. 1b and c). We suggest that the crystallisation reaction initiates with the formation of this poorly-ordered hydrated Ce-bearing carbonate phase, which transforms into crystalline Ce carbonates within the first 24 hours, in line with previous research by Rodriguez-Blanco *et al.*^57^ who reported that this same poorly-ordered precursor phase had a lifetime of ∼20 minutes at 21 °C. Also, our findings are in agreement with the homogeneous nucleation experiments performed by Vallina *et al.*,^47,58^ who suggested a similar crystallisation sequence in other REE-bearing carbonates (La, Nd, Dy) also including a metastable poorly-ordered precursor phase. No amorphous Ce-carbonate was observed in our replacement experiments, but its occurrence may have gone unnoticed as a consequence of its short lifetime at our experimental temperatures tested. It is well-known that the formation of metastable amorphous precursors requires a rapid increase in the supersaturation levels of the aqueous solutions.^59,60^ The increase of supersaturation of the solution during the dissolution of the host grains is lower compared to homogeneous nucleation experiments and this would hinder the formation of transient poorly-ordered precursor phases. However, the supersaturation levels can rapidly increase in the solid–solution interphase, close to the dissolving surface of the carbonate-bearing grains, thus, the formation of amorphous Ce-carbonate cannot be discarded. Its formation would depend on the dissolution rate of the host mineral during the earliest stages of the interaction processes.

### 4.2. Ce carbonate transient phases and cerianite

The general crystallisation evolution is consistent with a progressive dehydration sequence and agrees with the sequence identified by Vallina *et al.*^47,58^ and Szucs *et al.*^55,56^ The crystallisation sequence observed, proceeds through multiple transient (metastable) phases, prior to reaching the most insoluble and thermodynamically stable polymorph, with a progressive loss of structural water. This phenomenon is also known as the “Ostwald's Rule of Stages”, observed in many mineral systems (*e.g.*, Bots *et al.*;^61^ Szucs *et al.*;^55,56^ Van Driessche *et al.*^62^). We suggest the experiments evolve *via* three main steps during the replacement reactions. In step one, the interaction of host carbonate and the Ce-bearing solution produces REE-carbonates through the following chemical reactions:

Calcite/aragonite transformation into lanthanite:3CaCO_3(s)_ + 2Ce^3+^_(aq)_ + 8H_2_O → Ce_2_(CO_3_)_3_·8H_2_O_(s)_ + 3Ca^2+^_(aq)_

Dolomite producing lanthanite:3CaMg(CO_3_)_2(s)_ + 4Ce^3+^_(aq)_ + 8H_2_O → 2Ce_2_(CO_3_)_3_·8H_2_O_(s)_ + 3Ca^2+^_(aq)_ + 3Mg^2+^_(aq)_

Calcite or aragonite producing kozoite or hydroxylbastnasite:CaCO_3(s)_ + Ce^3+^_(aq)_ + H_2_O → CeCO_3_OH_(s)_ + Ca^2+^_(aq)_ + H^+^_(aq)_

Dolomite producing kozoite or hydroxylbastnasite:CaMg(CO_3_)_2(s)_ + 2Ce^3+^_(aq)_ + H_2_O → 2Ce(CO_3_)OH_(s)_ + Ca^2+^_(aq)_ + Mg^2+^_(aq)_ + H^+^_(aq)_

In these reactions, the pH of the initial Ce-bearing solution is ∼5.1 which first increases due to the buffering effect of the carbonate host minerals to pH ∼7.1. Following, in step two, the reaction proceeds with the formation of cerianite and the release of CO_2_ and H^+^, *via* the following reaction:Ce^3+^CO_3_OH_(s)_ → Ce^4+^O_2(s)_ + CO_2(aq)_ + H^+^_(aq)_ + *e*^−^

The decarbonation^63^ and H^+^ production is translated into a drastic decrease in the solution pH and explains the observed bubbles (Fig. ESI-4a†) upon opening the hydrothermal reactors in the higher temperature experiments. The transformation reaction above is also a redox (oxidation) reaction as Ce^3+^ transforms to Ce^4+^ by losing one electron. As step three, we must consider that cerianite can also form directly replacing the carbonate host mineral without any previous formation of transient REE-carbonate phases as it was observed at higher temperatures (≥165 °C). This proceeds through the following reactions:

Calcite or aragonite directly producing cerianite:2CaCO_3(s)_ + Ce^3+^_(aq)_ → Ce^4+^O_2(s)_ + 2Ca^2+^_(aq)_ + 2CO_2(aq)_ + *e*^−^

Dolomite directly producing cerianite:CaMg(CO_3_)_2(s)_ + Ce^3+^_(aq)_ → Ce^4+^O_2(s)_ + Ca^2+^_(aq)_ + Mg^2+^_(aq)_ + 2CO_2(aq)_ + *e*^−^

The reaction initiates with the Ce-bearing solution, pH ∼5.1. The pH is expected to increase at the early stage of the reaction due to the dissolution of the carbonate host mineral, however, it decreases to acidic values as the consequence of the crystallisation of cerianite driven by the redox oxidation reaction from Ce^3+^ to Ce^4+^ and the decarbonation process. This reaction pathway occurs due to the boosting effect of the temperature.

In the cases of the solution experiments, each REE-carbonate phase and cerianite can form directly from the mixture of Ce-bearing and Na_2_CO_3_ solutions.

Lanthanite production from solution:3CO_3_^2−^_(aq)_ + 2 Ce^3+^_(aq)_ + 8H_2_O → Ce_2_(CO_3_)_3_·8H_2_O_(s)_

Kozoite production from solution:CO_3_^2−^_(aq)_ + Ce^3+^_(aq)_ + H_2_O → CeCO_3_OH_(s)_ + 2H^+^_(aq)_

Cerianite production from solution:2CO_3_^2−^_(aq)_ + Ce^3+^_(aq)_ → Ce^4+^O_2(s)_ + 2CO_2(g)_ + *e*^−^

The absence of Ce-hydroxylbastnasite in all solution experiments is a possible consequence of the experimental temperature tested. In our replacement experiments, we only observed hydroxylbastnasite at temperatures above 165 °C, in agreement with Szucs *et al.*^55,56^ who found La-, Pr-, Nd-hydroxylbastnasite forming only above that temperature. Our solution experiments were conducted at a maximum 80 °C. Thus, we cannot completely exclude the possibility of some hydroxylbastnasite forming directly from the solution at temperatures above 165 °C before Ce-kozoite transforms into cerianite. However, obtaining precipitates from my mixing pre-heated solutions at temperatures ≥100 °C is not feasible with our experimental setting. Also, cooling the reactors to <100 °C to obtain solid samples could potentially result in the formation of low-temperature occurring REE-carbonates during the cooling process, biasing the results.

**Fig. 15:**
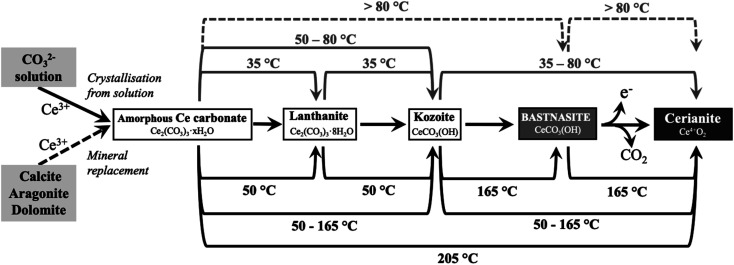
Reaction pathways toward cerianite (Ce^4+^O_2_) *via* Ce^3+^-bearing carbonates observed during solution experiments (top lines) and replacement experiments (bottom lines). Each line shows the temperature at which the reaction takes place. Continuous lines correspond to observed reactions, while dashed lines correspond to expected reactions that were not observed in our experiments.

In previous studies with single REE (La, Pr, Nd),^55,56^ hydroxylbastnasite was identified as the final step of the pathway. However, our experiment with Ce shows that the most stable and final phase of the crystallisation sequence is cerianite. The reaction pathway proceeds through Ce-bearing carbonates (Fig. 15), similar to the sequence observed by Szucs *et al.*^55,56^ However, it further proceeds and finalizes in cerianite (CeO_2_). This is consistent with previous studies by Janoš *et al.* (2017)^29^ who synthesized cerianite *via* homogeneous precipitation and found some Ce carbonates as intermediate phases. The decarbonation reaction triggered by the formation of cerianite is a consequence of the oxidation of Ce^3+^ to Ce^4+^ as it has a more stable atomic configuration. The electron configuration of metallic cerium is [Xe] 4f^1^5d^1^6 s^2^, of Ce^3+^ is [Xe] 4f^1^5d^0^6s^0^, while Ce^4+^ has an electron configuration [Xe] 4f^0^5d^0^6s^0^. As Ce^4+^ has the stable configuration of a nearest noble gas,^64,65^ it is more stable than Ce^0^, so Ce^3+^ will also tend to oxidize to Ce^4+^ to get the stable configuration of the nearest noble gas. The Eh–pH diagram of Ce, presented by Brookins (1988)^66^ shows that the initial pH evolution from 5.1 to more basic values due to the dissolution of the host carbonates will promote the oxidation of Ce^3+^ to Ce^4+^ in solution. This oxidation process is directly translated into the formation of Ce^4+^O_2_, and the dissolution–crystallisation of the already formed Ce carbonates (lanthanite, kozoite, hydroxylbastnasite) and the host Ca–Mg carbonate minerals. This crystallisation pathway comes to an end with the formation of cerianite, the most insoluble phase (log *K*_cer_ = −59.3 ± 0.3 in 0.01 M NaClO_4_)^67^ compared to the REE carbonates^46,68,69^ or the Ca–Mg carbonates (log *K*_ara_ = −8.34; log *K*_cal_ = −8.48; log *K*_dol_ = −18.14).^55,56,70,71^

As the system follows the “Ostwald's Rule of Stages”, the final pH can drop significantly once all the host carbonate crystals (Ca, Mg, and REE carbonates) are consumed as the consequence of the decarbonation during the crystallisation of cerianite. Calculations with PHREEQC show that the pH can drastically decrease to values close to ∼1.5 pH in the replacement experiment if all the host carbonates are consumed. The final pH might be less acidic if there is any excess amount of the host Ca–Mg carbonate available. This decarbonation during the final transformation from kozoite/hydroxylbastnasite or from the Ca–Mg carbonates to cerianite is also translated into an increase of the porosity of the pseudomorphs. The molar volume of cerianite (23.86 cm^3^ mol^−1^) is a lot smaller compared to kozoite (45.59 cm^3^ mol^−1^), hydroxylbastnasite (44.91 cm^3^ mol^−1^), and the divalent Ca–Mg carbonates, aragonite (34.17 cm^3^ mol^−1^), calcite (36.93 cm^3^ mol^−1^), and dolomite (64.12 cm^3^ mol^−1^).^72^ Therefore, the transformation of any of these carbonates into cerianite increases the porosity and the surface area of the final pseudomorph (Fig. 14). Upon mentioning molar volumes, we must note here during the earlier stages of the replacement of the host Ca–Mg carbonates by Ce carbonates, the larger molar volume of the newly formed solids (lanthanite, kozoite, hydroxylbastnasite) compared to the host could also result in the situation of partial equilibrium.^73,74^ The reactive solid (host grains) could become isolated from the aqueous solution by the secondary solids (Ce carbonates) forming a dense enough crust that would be impenetrable by the aqueous phase. In our experiments, we have not observed any indications of a partial equilibrium scenario as they resulted in full replacements of the host grains. This may be a consequence of the multiple growth defects observed in the newly formed Ce carbonate crystals (*e.g.*, Fig. 12, and 13a), which are a consequence of the rapid crystallisation kinetics. These defects would allow the solution to reach the Ca–Mg carbonate seed.

The crystallisation kinetics of the Ce carbonates is strongly dependent on the pH of the solution and the dissolution rates of aragonite, calcite, and dolomite.^55,56,70,71^ The fast dissolution of the host minerals is translated into a supersaturated solution with respect to Ce-bearing carbonates and cerianite even during the few minutes of reaction. The supersaturation levels can drastically increase in the host mineral–solution interface, leading to very rapid crystal nucleation of secondary REE-carbonates or cerianite. Aragonite, with the highest dissolution rate, will dissolve ≈10 times faster than dolomite at the same starting pH (5.1). This means that the crystallisation reactions will proceed the fastest in the aragonite system, followed by the bit slower calcite and dolomite. Therefore, the dissolution of only a few monolayers of the surface of the host mineral can be translated into the formation of a fluid boundary layer near-surface of the host seed, resulting in the development of a surface precipitate.^75^ As this surface precipitate forms, more carbonate ions are removed from the aqueous solution, enhancing the dissolution of the host grain, and increasing the growth rate of the overgrowth. In particular, our observations of spherulitic Ce carbonates in the replacement experiments (*e.g.*, Fig. 5, 6, 12, 13a) indicate very rapid nucleation kinetics which requires high supersaturation levels (SI > 2–3).^76–78^ Calculations with PHREEQC show that the SI values are always high enough in our experiments to promote this mechanism of crystallisation (Tables ESI-4 and 5†). For example, upon mixing the 50 mM Na_2_CO_3_ and 50 mM Ce(NO_3_)_3_ in the solution experiments, the SI value of Ce-lanthanite is 11.28 and 16.8 for cerianite at 50 °C (pH ∼ 7.2), while at 80 °C the SI value of Ce-lanthanite is 11.03 and for cerianite is 15.41 (pH ∼ 6.7). In the replacement experiments, equilibrating the 50 mM Ce-bearing aqueous solution with respect to calcite at 50 °C results in a SI of 9.93 for Ce-lanthanite and 15.88 for cerianite (pH ∼ 6.7), while at 90 °C these SI slightly decrease to 8.56 for Ce-lanthanite and 14.02 for cerianite (pH ∼ 5.95). Unfortunately, there is no information for pure Ce-kozoite or Ce-bastnasite as their solubility products have not been determined. However, Ce-kozoite or Ce-bastnasite are less soluble phases compared to Ce-lanthanite (in accordance with the “Ostwald's Rule of Stages”) meaning that the SI values of Ce-kozoite and Ce-bastnasite should be higher than Ce-lanthanite. Also, it must be noted that even in the case of a Ce-lanthanite-saturated solution, the SI value of kozoite would be high enough to promote spherulitic growth (as seen by Vallina *et al.*,^47^ for La carbonates).

As the control experiments at ≥165 °C resulted in cerianite precipitate, it must be discussed whether the formation of cerianite in the experiments is triggered by the presence of carbonate ions, or it is a spontaneous reaction occurring during the dissociation of Ce(NO_3_)_3_·6H_2_O in solution. Two evidences from our results need to be highlighted here: (1) Ce-carbonate always precipitates prior to the formation of cerianite. In the solution experiments, the formation of amorphous Ce carbonate is instantaneous, followed by the formation of crystalline Ce carbonates in less than 20 minutes (Rodriguez-Blanco *et al.*,^57^), and lastly by cerianite. Similarly, in the replacement experiments, the formation of Ce carbonates always happens prior to the crystallisation of cerianite, except at 205 °C where we could be uncertain whether any Ce carbonates form at all as the reaction proceeds too rapidly at such a high temperature. However, this leads us to the second noteworthy evidence: (2) cerianite was always identified in association with carbonates on their surfaces. At temperatures below 205 °C cerianite always crystallised on the surface of previously formed Ce carbonates. At 205 °C, no Ce carbonates were identified, but cerianite nucleated on the surface of the host Ca–Mg carbonate (calcite/dolomite/aragonite), forming surface precipitates and preserving the shape of the host grain during the replacement reactions. If cerianite had nucleated directly from the aqueous solution at the very early stages of the reaction, it would had formed prior to Ce carbonates, potentially depleting Ce from the aqueous solution before the crystallisation of the Ce carbonates. Also, if cerianite had nucleated directly from solution in our experiments, it would have been found as a massive precipitate in the reactor instead of replacing the carbonate minerals. Therefore, we suggest that the surface precipitation of cerianite is a consequence of the Ce^3+^ → Ce^4+^ oxidation process (favoured at high temperatures) that is locally triggered by the more basic pH at the surface–solution interface of the carbonate minerals.

## 5. Implications for natural systems and industry

Our findings shed new light on the conditions and crystallisation mechanisms in which cerianite has a key role in nature and the laboratory. The results of this study open a new path to understanding the mineralogy and chemistry of several rare earth deposits. Naturally formed cerianite has been found: (1) in association with bastnasite/monazite;^31–36^ (2) as a secondary mineral, as a result of hydrothermally-altered carbonate/apatite weathering;^2,31,37,38^ (3) replacing early crystallised ancylite and fluorocarbonates;^40^ (4) as an overgrowth on Ce-depleted monazite.^40^ Our experiments explain these findings in natural deposits and are consistent with the decarbonation processes of rare earth carbonates due to the crystallisation of Ce-carbonates under low-hydrothermal conditions (35–205 °C). Consequently, Ce-rich carbonates will tend to be unstable in carbonate deposits with large concentrations of Ce under hydrothermal conditions.

Our findings also provide fundamental information for cerianite and Ce carbonate synthesis. Cerianite has various industrial applications (*e.g.*, catalysis,^5,9,10^ biomedical research^11,16–22^), in-demand from quantum,^27,79^ nano-scale,^8,25,80^ and microscale,^81,82^ produced with various methods.^19,23–26^ Our methodology allows a facile, environmental-friendly and cost-efficient synthesis of Ce carbonate and Ce oxides. The findings serve as a baseline for targeted Ce-bearing functional material synthesis (Table ESI-1†).

## 6. Conclusion

The interaction of Ce-rich fluids with carbonates ions or with carbonate minerals results in the formation of cerianite *via* a multi-step crystallisation pathway: amorphous Ce carbonate → Ce-lanthanite → Ce-kozoite → Ce-hydroxylbastnasite → cerianite. The reaction was found to be strongly governed by the temperature and the solubility of the Ca–Mg carbonates. The higher the temperature, the faster cerianite crystallisation is promoted, decreasing the number of intermediate steps of this crystallisation pathway. The solubility of the Ca–Mg carbonates affects the supersaturation levels and thus, the crystal size and morphology of the newly formed phases. Our results show that the formation of cerianite has a strong effect on mineralisation reactions occurring in Ce-rich deposits, promoting decarbonation processes and increasing the porosity of the host replaced minerals. This knowledge of the reaction pathways and kinetics can be used to synthesise targeted materials with tailored structures and chemistries for industrial purposes.

## Conflicts of interest

There are no conflicts to declare.

## Supplementary Material

RA-013-D3RA00519D-s001
