# Supplementary material for: The role of nanocerianite (CeO2) in the stability of Ce carbonates at low-hydrothermal conditions
Source: RSC Adv. 2023 Feb 28;13(10):6919–35. doi: 10.1039/d3ra00519d (PMC9972569; doi:10.1039/d3ra00519d)
Supplement: RA-013-D3RA00519D-s001 [file RA-013-D3RA00519D-s001.pdf]

Electronic Supplementary Material (ESI) for RSC Advances.
This journal is © The Royal Society of Chemistry 2023

Supplementary information

The role of nanocerianite (CeO_2) in the stability of Ce carbonates at low-hydrothermal conditions.

Adrienn Maria Szucs[a], Melanie Maddin [a], Daniel Brien [a], Remi Rateau [a] and Juan Diego Rodriguez-Blanco[b]

[a] Department of Geology, School of Natural Sciences, Trinity College Dublin, Ireland.

[b] iCRAG, Department of Geology, School of Natural Sciences, Trinity College Dublin, Ireland.

Table SI-1. Summary of comparative results on a selection of previous and present literatures on REE carbonates and cerianite characterisation and synthesis methods, highlighting the novelty of this study.

Publication	Experimental approach	Focus	Produced Materials	Major Impact
**This study**	**Solution and replacement experiments**	**Ce-bearing solution interaction with carbonate ions studied via solution experiments and replacement experiments. An investigation of the crystallisation pathway.**	**amorphous Ce carbonate; Ce-lanthanite; Ce-kozoite; Ce-hydroxylbasnasite; Cerianite**	– **Understanding the formation of cerianite in natural deposits at low-hydrothermal conditions.**<br>– **Ce-carbonates and cerianite synthesis with various morphologies and size**
Szucs et al., 2022 [1]	Replacement experiments	Dolomite or aragonite seed interaction with La, Pr, Nd, or Dy- bearing solutions	La-lanthanite; La-kozoite, La-hydroxylbastnasite; Pr-lanthanite; Pr-kozoite; Pr-hydroxylbastnasite; Nd-lanthanite; Nd, kozoite; Nd-hydroxylbasnasite; Dy-tengerite; Dy-kozoite	– Understanding the formation of bastnasite in natural deposits and the influence of carbonate mineral involved at low-hydrothermal conditions.
Szucs et al., 2021 [2]	Replacement experiments	Calcite seed interaction with La, Nd or Dy-bearing solutions	La-lanthanite; La-kozoite; La-hydroxylbastnasite; Nd-lanthanite; Nd-kozoite; Nd-hydroxylbasnasite; Dy-kozoite	– Revealed the formation of bastnasite in natural deposits and the effect of temperature and ionic radii of the REE in question at low-hydrothermal conditions.
Janoš et al., 2017 [3]	Solution experiment	Investigating cerianite's ability to breakdown biologically relevant organophosphates	Nanocrystalline cerianite	– Cerianite's unusual phosphatase-mimetic ability was demonstrated
Voigt et al., 2016 [4]	Solution experiments	Investigation of hydroxylbastnasite solubility in aqueous solutions at 25 °C	Nd- and La-hydroxylbastnasite; Nd-kozoite	– solubility products (K_{SP}) of Nd- and La-hydroxylbastnasite and Nd-kozoite were defined
Vallina et al., 2015 [5]	Solution experiments	The role of amorphous precursors in the formation of La and Nd carbonates	Amorphous La-lanthanum carbonate; La-dioxycarbonate; La-oxide; La-lanthanite; La-kozoite; La-hydroxylbastnasite; Amorphous Nd-lanthanum carbonate Nd-dioxycarbonate; Nd-oxide; Nd-lanthanite;Nd-tengerite; Nd-kozoite; Nd-hydroxylbastnasite	– Fabrication of targeted La and Nd carbonate synthesis.
Ravishankar et al., 2015 [6]	Solution combustion method	Investigating cerianite for photocatalytic and antibacterial activity	Cerianite	– Particle characterisation and revealed photocatalytic and antibacterial activity

Vallina et al., 2014 [7]	Solution experiments	Effect of heat on the formation of hexagonal $NdCO_3OH$	Amorphous Nd- hydroxycarbonate; Nd-hydroxycarbonate	– Amorphous Nd- hydroxycarbonate and Nd-hydroxycarbonate fabrication with different sizes and morphologies by temperature control.
Rodriguez-Blanco et al., 2014 [8]	Solution experiments	Investigating the role of La, Ce, Pr and Nd ions in the crystallisation of lanthanites	La, Ce, Pr, Nd amorphous carbonate precursor; La, Ce, Pr, Nd-lanthanite	– The differences in ionic potential and in dehydration energy of the La, Ce, Pr and Nd ions control the kinetics of the lanthanite formation.
Vallina et al., 2013 [9]	Solution experiments	Dy carbonate characterisation	Amorphous Dy carbonate; Dy-kozoite	– Amorphous Dy carbonate and Dy-kozoite fabrication with different sizes and morphologies by temperature control.
Suresh et al. 2013[10]	Solution experiment	Investigating optical and electrical properties of nano cerianite formed from solution at room temperature.	Nano cerianite	– Determined the activation energy of their material.
Zhang et al., 2009 [12]	Sol-gel method	Method development for quantum-size cerianite synthesis	Cerianite	– A sol-gel method for nano-size cerianite synthesis
Sathyamurthy et al., 2005 [13]	Reverse micellar synthesis	Method development for cerianite synthesis	Cerianite nano particles	– Cerianite reverse micellar synthesis was demonstrated.
Özer 2001 [14]	Sol-gel method	Investigating the optical properties and electrochromic of sol-gel deposited ceria film	Ceria (CeO_2) films	– Determined optical properties of sol–gel spin coated ceria films.
Chai and Mroczkowski 1978 [15]	Hydrothermal method using rare earth carbonate hydrate as starting material	Y, La, Gd and Er carbonate formation at temperature range 250-500 °C and 1 to 2 kb conditions.	Y, La, Gd and Er carbonate	– Ionic radii of the Y, La, Gd and Er ions influence the thermal stability of the rare earth carbonate. Crystal morphology is controlled by temperature, solution chemistry, ionic radii.
Kozo et al., 1973[16]	Solution experiments	Investigation of rare earth carbonates' crystal parameters	The materials were labelled the following way: lanthanite-type $Ln_2(CO_3)_3·8H_2O$ (Ln=La, Ce), tengeritetype $Ln_2(CO_3)_3·nH_2O$ (Ln=Nd, Sm, Gd, Dy, Ho, Er, and Y, n=2–3), monoxocarbonate-type $Ln_2O(CO_3)_2·nH_2O$ (Ln=La, Ce, Nd, and Sm, n=1–2) and a hydrated double carbonate of rare earth and sodium (rare earth= La, Ce, Nd, Sm, Gd, Dy, and Y)	– Defined crystal parameters by X-ray diffraction
Graham 1955 [17]	The discovery of Cerianite	The first characterisation of cerianite	Cerianite	Cerianite was characterized the first time.

Table SI-2. Details of FTIR stretching (ν) and bending (δ)-vibrational band assignments and corresponding references for the water and carbonate species in the poorly-ordered hydrated Ce carbonate, with band numbers corresponding to those shown in the spectra in Figure 1b.

Band Number	Mode of vibration	Bibliography
1	v (O-H)	[18]
2	δ (O-H)	[19,20]
3	v_3 asym. CO_3	[21]
4	v_3 asym. CO_3	[22]
5	v_1 sym. CO_3	[23,24]
6	v_2 asym. CO_3	[24]
7	v_4 asym. CO_3	[24,25]
8	v_4 CO_3	[23,24]

Table SI-3. Unit cell parameters of the original calcite, dolomite, aragonite, and the Ce-bearing carbonate minerals and cerianite crystallised in the experiments.

**Mineral**	**Space group**	**Solution Experiments**	**Replacement experiments**		
			Calcite	Dolomite	Aragonite
Calcite	$R\bar{3}c$	–	a = b = 4.989(17) Å<br>c = 17.062(55) Å<br>vol = 367.78(28)Å^3	–	–
Dolomite	$R\bar{3}$	–	–	a = b = 4.8028(13) Å<br>c = 15.9998(43) Å<br>vol = 319.62(20) Å^3	–
Aragonite	Pmnc	–	–	–	a = 4.9608(13) Å<br>b = 7.9663(20)Å<br>c = 5.7421(15) Å<br>vol = 229.92(10) Å^3
Ce-lanthanite	Pbnb	a = 9.498(11)Å<br>b = 16.945(20) Å<br>c = 8.925(11) Å<br>vol = 1436.4(30) Å^3	–	–	a = 9.5083 Å<br>b = 16.9563 Å<br>c = 8.92280 Å<br>vol = 1438.5949 Å^3
Ce-kozoite	Pnma	a = 7.3199(15) Å<br>b = 5.0090(10) Å<br>c = 8.55702(18) Å<br>vol = 313.50(11) Å^3	a = 7.3144(18) Å<br>b = 5.000(12) Å<br>c = 8.5457(20) Å<br>vol = 312.55(13) Å^3	a = 7.3176(14) Å<br>b = 5.0044(95) Å<br>c = 8.5490(16) Å<br>vol = 312.28(50)Å^3	a = 7.3192(57) Å<br>b = 5.0103(37) Å<br>c = 8.5697(56) Å<br>vol = 314.26(39) Å^3
Ce-hydroxylbastnasite	$P\bar{6}$	–	a = b = 12.3396(30) Å<br>c = 9.9035(27) Å<br>vol = 1305.94(73)Å^3	a = b = 12.5301(24) Å<br>c = 9.9732(10) Å<br>vol = 1356.05(58) Å^3	a = b = 12.5371(54) Å<br>c = 9.96187(72) Å<br>vol = 1356.01(15) Å^3
Cerianite	$Fm\bar{3}m$	a = b = c = 5.4200(11) Å<br>vol = 159.216(97) Å^3	a = b = c = 5.4150(16) Å<br>vol = 158.78(14) Å^3	a = b = c = 5.4147(27) Å<br>vol = 158.749(24) Å^3	a =b = c = 5.4133(69) Å<br>vol = 158.64(30) Å^3

Table SI-4. pH and saturation indices for Ce-lanthanite and cerianite calculated with PHREEQC in the early stages of the replacement reactions, when calcite/dolomite/aragonite are equilibrated with 50 mM $Ce(NO_3)_3$-bearing aqueous solutions.

	Calcite		Dolomite		Aragonite	
**Temperature**	**pH**	**Phases: SI**	**pH**	**Phases: SI**	**pH**	**Phases: SI**
50 °C	6.68	Ce-lanthanite: 9.93	6.55	Ce-lanthanite: 9.33	6.74	Ce-lanthanite: 10.12
		Cerianite: 15.88		Cerianite: 15.59		Cerianite: 15.99
90 °C	5.95	Ce-lanthanite: 8.56	5.81	Ce-lanthanite: 7.72	5.99	Ce-lanthanite: 8.81
		Cerianite: 14.02		Cerianite: 13.71		Cerianite: 14.11
165 °C	5.2	Ce-lanthanite: 2.93	5	Ce-lanthanite:2.01	5.27	Ce-lanthanite: 3.17
		Cerianite: 11.28		Cerianite: 10.94		Cerianite: 11.36
201 °C	5.01	Ce-lanthanite: -0.56	4.84	Ce-lanthanite: -1.40	5.13	Ce-lanthanite: -0.35
		Cerianite: 10.18		Cerianite: 9.88		Cerianite: 10.26

Table SI-5. pH and saturation indices for Ce-lanthanite and cerianite calculated with PHREEQC in the early stages of the solution experiments after mixing 50mM Na_2CO_3 and 50mM $Ce(NO_3)_3$ aqueous solutions.

Temperature	pH	Phases: SI
35 °C	7.47	Ce-lanthanite: 11.29
		Cerianite: 17.41
50 °C	7.21	Ce-lanthanite: 11.28
		Cerianite: 16.80
80 °C	6.67	Ce-lanthanite: 11.03
		Cerianite: 15.41

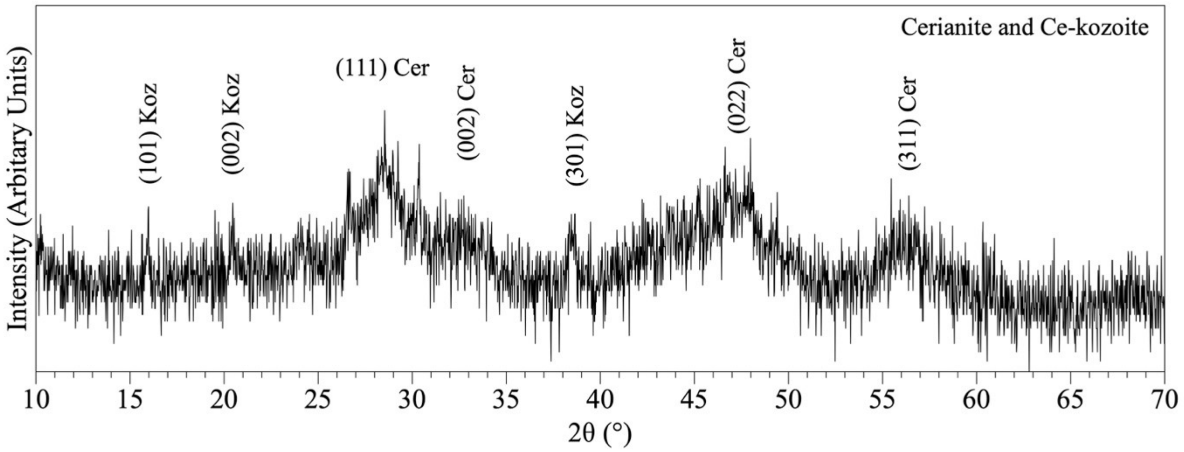

Figure SI-1. XRD pattern of nano-crystalline cerianite and Ce-kozoite formed after 3 hours in the solution experiments after mixing 10ml of 15mM $Ce(NO_3)_3$ and 10ml of 20 mM Na_2CO_3 pre-heated at 80 °C.

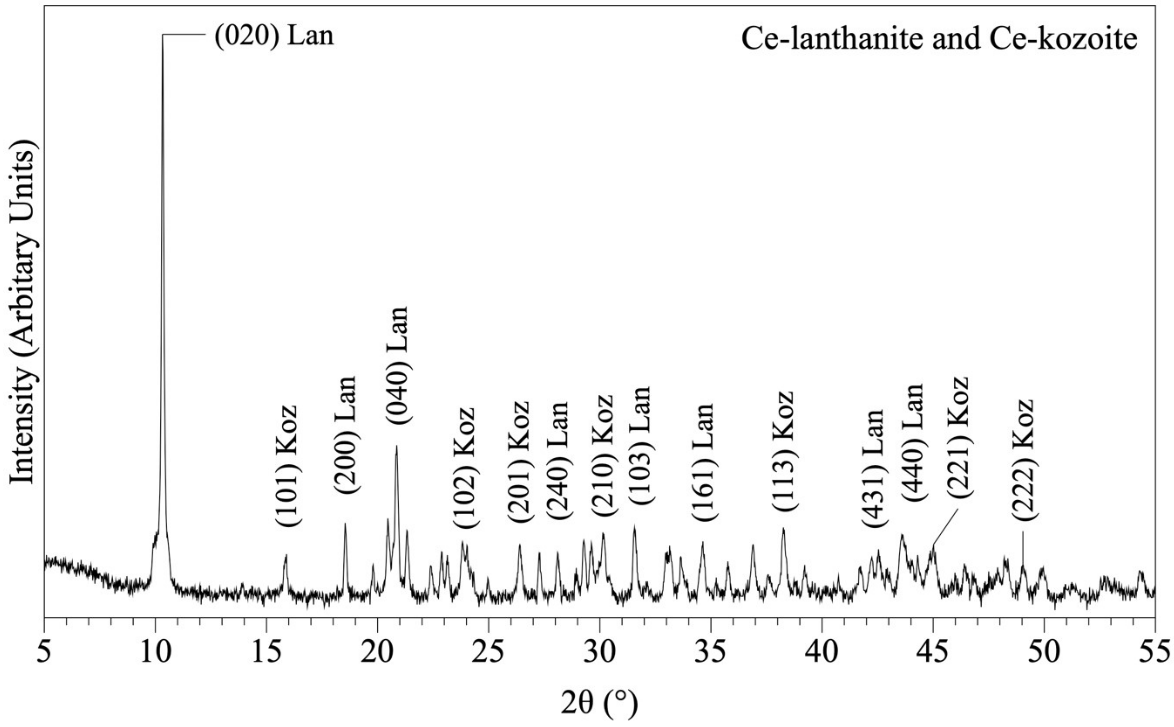

Figure SI-2. XRD pattern of cerianite and Ce-kozoite formed after 1 day of reaction in the solution experiments after mixing 10ml of 50mM $Ce(NO_3)_3$ and 10ml of 50 mM Na_2CO_3 pre-heated at 35 °C.

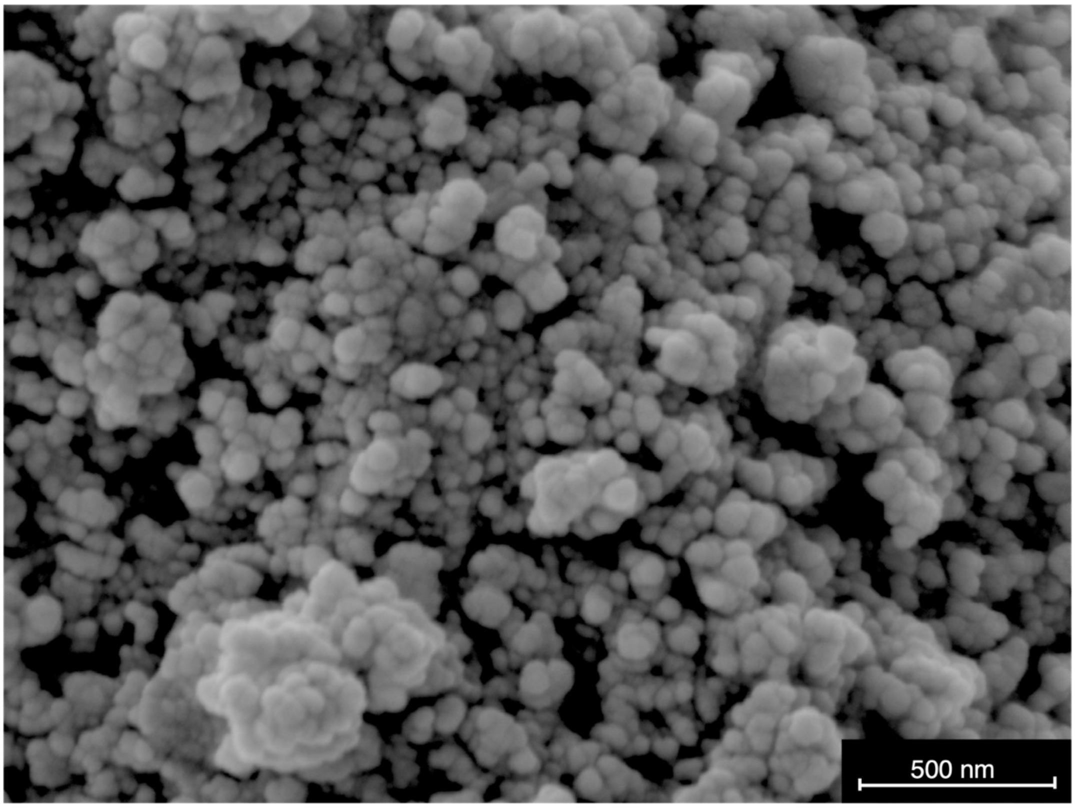

Figure SI-3. SEM image of nano-crystalline cerianite formed after 3 hours in the solution experiments after mixing 10ml of 15mM $Ce(NO_3)_3$ and 10ml of 20 mM Na_2CO_3 pre-heated at 80 °C.

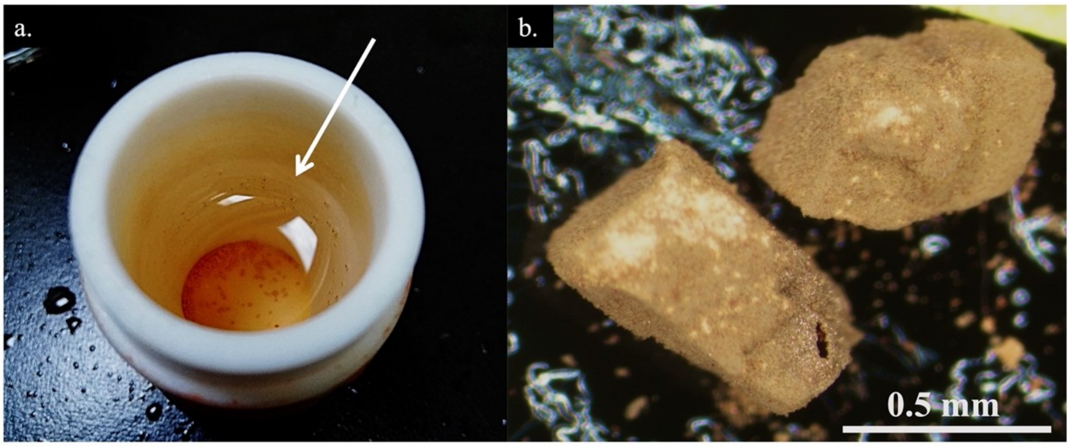

Figure-SI-4. a) image of gas bubbles seen on the side of the Teflon reactor when sampling the experiment that contained dolomite reacted for 3 days at 165 °C and b) a closer image of the dry material.

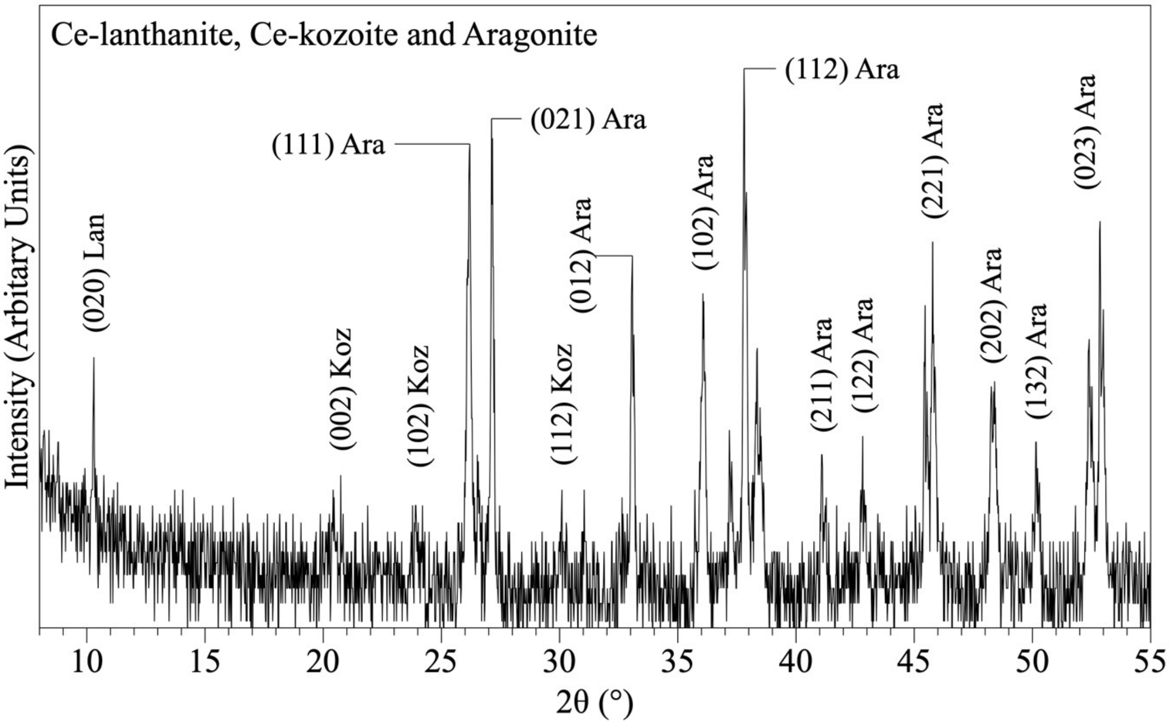

Figure SI-5. XRD pattern of sample obtained in the aragonite replacement experiment at 50 ºC after 1 day of reaction, showing the formation of Ce-lanthanite and Ce-kozoite.

Supplementary Information References

[1] A.M. Szucs, M. Maddin, D. Brien, P.C. Guyett, J.D. Rodriguez-Blanco, Targeted Crystallization of Rare Earth Carbonate Polymorphs at Hydrothermal Conditions via Mineral Replacement Reactions, Glob. Challenges. n/a (2022) 2200085. https://doi.org/https://doi.org/10.1002/gch2.202200085.

[2] A.M. Szucs, A. Stavropoulou, C. O'Donnell, S. Davis, J.D. Rodriguez-Blanco, Reaction Pathways toward the Formation of Bastnäsite: Replacement of Calcite by Rare Earth Carbonates, Cryst. Growth Des. 21 (2021). https://doi.org/10.1021/acs.cgd.0c01313.

[3] P. Janoš, J. Henych, J. Pfeifer, N. Zemanová, V. Pilařová, D. Milde, T. Opletal, J. Tolasz, M. Malý, V. Štengl, Nanocrystalline cerium oxide prepared from a carbonate precursor and its ability to breakdown biologically relevant organophosphates, Environ. Sci. Nano. 4 (2017). https://doi.org/10.1039/c7en00119c.

[4] M. Voigt, J.D. Rodriguez-Blanco, B. Vallina, L.G. Benning, E.H. Oelkers, An experimental study of hydroxylbastnasite solubility in aqueous solutions at 25°C, Chem. Geol. 430 (2016) 70–77. https://doi.org/https://doi.org/10.1016/j.chemgeo.2016.03.012.

[5] B. Vallina, J.D. Rodriguez-Blanco, A.P. Brown, J.A. Blanco, L.G. Benning, The role of amorphous precursors in the crystallization of La and Nd carbonates, Nanoscale. 7 (2015) 12166–12179. https://doi.org/10.1039/C5NR01497B.

[6] T.N. Ravishankar, T. Ramakrishnappa, G. Nagaraju, H. Rajanaika, Synthesis and Characterization of CeO2 Nanoparticles via Solution Combustion Method for Photocatalytic and Antibacterial Activity Studies., ChemistryOpen. 4 (2015) 146–154. https://doi.org/10.1002/open.201402046.

[7] B. Vallina, J.D. Rodriguez-Blanco, J.A. Blanco, L.G. Benning, The effect of heating

on the morphology of crystalline neodymium hydroxycarbonate, NdCO3OH, Mineral. Mag. 78 (2014) 1391–1397. https://doi.org/DOI: 10.1180/minmag.2014.078.6.05.
[8] J.D. Rodriguez-Blanco, B. Vallina, J.A. Blanco, L.G. Benning, The role of REE3+ in the crystallization of lanthanites, Mineral. Mag. 78 (2014) 1373–1380. https://doi.org/DOI: 10.1180/minmag.2014.078.6.03.
[9] B. Vallina, J. Rodriguez-Blanco, A. Brown, J. Blanco, L. Benning, Amorphous dysprosium carbonate: Characterization, stability, and crystallization pathways, J. Nanoparticle Res. 15 (2013) 1438–1450. https://doi.org/10.1007/s11051-013-1438-3.
[10] R. Suresh, V. Ponnuswamy, R. Mariappan, Effect of annealing temperature on the microstructural, optical and electrical properties of CeO2 nanoparticles by chemical precipitation method, Appl. Surf. Sci. 273 (2013) 457–464. https://doi.org/https://doi.org/10.1016/j.apsusc.2013.02.062.
[11] T. Roncal-Herrero, J.D. Rodríguez-Blanco, E.H. Oelkers, L.G. Benning, The direct precipitation of rhabdophane (REEPO4·nH2O) nano-rods from acidic aqueous solutions at 5–100 °C, J. Nanoparticle Res. 13 (2011) 4049–4062. https://doi.org/10.1007/s11051-011-0347-6.
[12] Q.-C. Zhang, Z.-H. Yu, G. Li, Q.-M. Ye, J.-H. Lin, Synthesis of quantum-size cerium oxide nanocrystallites by a novel homogeneous precipitation method, J. Alloys Compd. 477 (2009) 81–84. https://doi.org/https://doi.org/10.1016/j.jallcom.2008.10.059.
[13] S. Sathyamurthy, K.J. Leonard, R.T. Dabestani, M.P. Paranthaman, Reverse micellar synthesis of cerium oxide nanoparticles, Nanotechnology. 16 (2005). https://doi.org/10.1088/0957-4484/16/9/089.
[14] N. Özer, Optical properties and electrochromic characterization of sol–gel deposited ceria films, Sol. Energy Mater. Sol. Cells. 68 (2001) 391–400. https://doi.org/https://doi.org/10.1016/S0927-0248(00)00371-8.
[15] B.H.T. Chai, S. Mroczkowski, Synthesis of rare-earth carbonates under hydrothermal conditions, J. Cryst. Growth. 44 (1978) 84–97. https://doi.org/https://doi.org/10.1016/0022-0248(78)90331-7.
[16] K. Nagashima, H. Wakita, A. Mochizuki, The Synthesis of Crystalline Rare Earth Carbonates, Bull. Chem. Soc. Jpn. 46 (1973) 152–156. https://doi.org/10.1246/bcsj.46.152.
[17] A.R. Graham, Cerianite Ceo2: A new rare-earth oxide mineral, Am. Mineral. 40 (1955) 560–564.
[18] G.H. Cartledge, Studies on the periodic system II. The ionic potential and related properties 1, J. Am. Chem. Soc. 50 (1928) 2863–2872. https://doi.org/10.1021/ja01398a002.
[19] H. Gamsjäger, Solubility equilibria: From chemical potentiometry to industrial applications, Pure Appl. Chem. 67 (1995) 535–542.
[20] H. Niu, Q. Min, Z. Tao, J. Song, C. Mao, S. Zhang, Q. Chen, One-pot facile synthesis and optical properties of porous La2O2CO3 hollow microspheres, J. Alloys Compd. 509 (2011) 744–747.
[21] B. Bakiz, F. Guinneton, M. Arab, A. Benlhachemi, J.-R. Gavarria, Elaboration, characterization of LaOHCO3, La2O2CO3 and La2O3 phases and their gas solid interactions with CH4 and CO gases, Moroccan J. Condens. Matter. 12 (2010).
[22] M.J. Norman, J.E. Andrew, T.H. Bett, R.K. Clifford, J.E. England, N.W. Hopps, K.W. Parker, K. Porter, M. Stevenson, Multipass reconfiguration of the HELEN Nd: glass laser at the Atomic Weapons Establishment, Appl. Opt. 41 (2002) 3497–3505.
[23] D.J. Tobler, S. Shaw, L.G. Benning, Quantification of initial steps of nucleation and growth of silica nanoparticles: An in-situ SAXS and DLS study, Geochim. Cosmochim. Acta. 73 (2009) 5377–5393.

[24] A. V Radha, T.Z. Forbes, C.E. Killian, P. Gilbert, A. Navrotsky, Transformation and crystallization energetics of synthetic and biogenic amorphous calcium carbonate, Proc. Natl. Acad. Sci. 107 (2010) 16438–16443.
[25] G.P. Hatch, Dynamics in the global market for rare earths, Elements. 8 (2012) 341–346.
